# Understanding the evolution of phenotypical characters in the *Micarea prasina* group (Pilocarpaceae) and descriptions of six new species within the group

**DOI:** 10.3897/mycokeys.57.33267

**Published:** 2019-07-31

**Authors:** Beata Guzow-Krzemińska, Emmanuël Sérusiaux, Pieter P.G. van den Boom, A. Maarten Brand, Annina Launis, Anna Łubek, Martin Kukwa

**Affiliations:** 1 University of Gdańsk, Faculty of Biology, Department of Plant Taxonomy and Nature Conservation, Wita Stwosza 59, PL-80-308 Gdańsk, Poland University of Gdańsk Gdańsk Poland; 2 Evolution and Conservation Biology Unit, University of Liège, Sart Tilman B22, B-4000 Liège, Belgium University of Liège Liege Belgium; 3 Arafura 16, NL-5691 JA Son, The Netherlands Unaffiliated Son Netherlands; 4 Klipperwerf 5, NL-2317 DX Leiden, The Netherlands Unaffiliated Leiden Netherlands; 5 Botany unit, Finnish Museum of Natural History, P.O. Box 7, FI-00014 University of Helsinki, Finland Finnish Museum of Natural History Helsinki Finland; 6 The Jan Kochanowski University in Kielce, Institute of Biology, Świętokrzyska 15, PL-25-406 Kielce, Poland The Jan Kochanowski University in Kielce Kielce Poland

**Keywords:** Ancestral state reconstruction, lichenised fungi, morphology, mtSSU rDNA, secondary metabolites, taxonomy

## Abstract

Six new *Micarea* species are described from Europe. Phylogenetic analyses, based on three loci, i.e. mtSSU rDNA, *Mcm7* and ITS rDNA and ancestral state reconstructions, were used to evaluate infra-group divisions and the role of secondary metabolites and selected morphological characters on the taxonomy in the *M. prasina* group. Two main lineages were found within the group. The *Micarea micrococca* clade consists of twelve species, including the long-known *M. micrococca* and the newly described *M. microsorediata*, *M. nigra* and *M. pauli*. Within this clade, most species produce methoxymicareic acid, with the exceptions of *M. levicula* and *M. viridileprosa* producing gyrophoric acid. The *M. prasina* clade includes the newly described *M. azorica* closely related to *M. prasina* s.str., *M. aeruginoprasina* sp. nov. and *M. isidioprasina* sp. nov. The species within this clade are characterised by the production of micareic acid, with the exception of *M. herbarum* which lacks any detectable substances and *M. subviridescens* that produces prasinic acid. Based on our reconstructions, it was concluded that the ancestor of the *M. prasina* group probably had a thallus consisting of goniocysts, which were lost several times during evolution, while isidia and soredia evolved independently at multiple times. Our research supported the view that the ancestor of *M. prasina* group did not produce any secondary substances, but they were gained independently in different lineages, such as methoxymicareic acid which is restricted to *M. micrococca* and allied species or micareic acid present in the *M. prasina* clade.

## Introduction

Traditionally, morpho-anatomical characters, together with secondary metabolites, have played an important role in the lichen classification (e.g. Brodo 1978, 1986; Lumbsch 1998). With the introduction of molecular data, powerful tools for reconstructing phylogenetic relationships have become available. Furthermore, molecular phylogenies can serve as a backbone for tracing the evolution of morphological and chemical characters by reconstructing their ancestral states. Such interpretations of character evolution usually open new perspectives to the evolutionary history (Lumbsch et al. 2006).

Secondary metabolites have been traditionally used in the taxonomy of lichens at different taxonomic levels, although their values have been questioned by many authors (Lumbsch et al. 2006; Leavitt et al. 2011; Lutsak et al. 2017). In many cases, molecular data do not correspond with the chemical variation and, therefore, the correlation between them has to be evaluated for each taxonomic group *de novo* (e.g. Goffinet and Miadlikowska 1999; Kroken and Taylor 2001; Molina et al. 2004; Divakar et al. 2005, 2006; Elix et al. 2009; Buschbom and Mueller 2006; Nelsen and Gargas 2008; Nelsen et al. 2008; Lendemer et al. 2015; Ossowska et al. 2018). Moreover, the production of certain secondary metabolites might be triggered by the environment (e.g. climate, edaphic factors, associated symbionts) (Spribille et al. 2016; Lutsak et al. 2017).

The genus *Micarea* Fr., comprising ca. 100 species, is a cosmopolitan group of lichens which has been extensively studied in Europe by Coppins (1983) and Czarnota (2007). Phenotypical diversity in this group of lichens is not limited to morphological characters, but also includes diverse secondary metabolites and, hence, chemical variation plays an important role in their taxonomy. Recently, *Micarea* has received more attention and numerous species have been described based on anatomical, morphological and chemical characters and, in some cases, also molecular data (e.g. Czarnota and Guzow-Krzemińska 2010; Svensson and Thor 2011; Cáceres et al. 2013; Aptroot and Cáceres 2014; Brand et al. 2014; van den Boom and Ertz 2014; Guzow-Krzemińska et al. 2016; McCarthy and Elix 2016; van den Boom et al. 2017; Kantvilas 2018; Launis et al. 2019a, b).

Species delimitation within *Micarea* has been especially difficult in the *M. prasina* group which was first characterised by Coppins (1983) based on morphological, anatomical and chemical features. At first, the group included *M. prasina* Fr., the type species of the genus, as well as *M. hedlundii* Coppins and *M. levicula* (Nyl.). Coppins (1983) also suggested that *M. misella* (Nyl.) Hedl., *M. melanobola* (Nyl.) Coppins and *M. synotheoides* (Nyl.) Coppins might be related to *M. prasina*; however, as supported by recent molecular studies, *M. misella* and *M. synotheoides* do not belong to this group (Czarnota and Guzow-Krzemińska 2010; van den Boom et al. 2017; Launis et al. 2019a). *Micarea melanobola* was synonymised with *M. prasina* (Czarnota 2007), but recently found to be a distinct species (Launis et al. 2019b).

Coppins (1983) treated *M. prasina* in a wide sense including specimens with variable morphology and chemistry, which later were distinguished as distinct species, i.e. *M. micrococca* (Körb.) Gams ex Coppins for the methoxymicareic acid chemotype, *M. prasina* s.str. for the micareic acid chemotype and *M. subviridescens* (Nyl.) Hedl. for the prasinic acid chemotype (Coppins 2009). Further studies showed even higher chemical variation within the *M. prasina* group and *M. xanthonica* Coppins & Tønsberg with xanthones (thiophanic acid with satellites) and *M. viridileprosa* Coppins & van den Boom containing gyrophoric acid (Coppins and Tønsberg 2001; van den Boom and Coppins 2001) were recognised. Later, more new species were discovered, such as *M. nowakii* Czarnota & Coppins, *M. soralifera* Guzow-Krzemińska, Czarnota, Łubek & Kukwa and *M. meridionalis* van den Boom, Brand, Coppins & Sérus. producing micareic acid. Moreover, *M. byssacea* (Th. Fr.) Czarnota, Guzow-Krzemińska & Coppins, *M. czarnotae* Launis, van den Boom, Sérusiaux & Myllys, *M. laeta* Launis & Myllys, *M. microareolata* Launis, Pykälä & Myllys and *M. pseudomicrococca* Launis & Myllys containing methoxymicareic acid, as well as *M. tomentosa* Czarnota & Coppins and *M. herbarum* Brand, Coppins, Sérus. & van den Boom lacking any lichen substances detectable by thin layer chromatography (TLC), were added to this group (Czarnota 2007; Czarnota and Guzow-Krzemińska 2010; Guzow-Krzemińska et al. 2016; van den Boom et al. 2017; Launis et al. 2019a). These species were described, based on phenotypic characters and molecular data. Recently crystalline granules studied in polarised light were also presented as a novel species-level character for *Micarea* spp. (Launis et al. 2019b). During the preparation of the final version of this paper, several other species within *M. prasina* group have also been described (Launis et al. 2019b), but those have not been included in our analyses.

Moreover, several other new species likely to belong to the *M. prasina* group have been described. Two such species were described from Réunion, i.e. *M. melanoprasina* Brand, van den Boom & Sérus. producing a substance probably related to micareic acid and *M. hyalinoxanthonica* Brand, van den Boom & Sérus. containing a xanthone (probably thiophanic acid) (Brand et al. 2014). Furthermore, one species was described from Brazil, i.e. *M. corallothallina* M. Cáceres, D. A. Mota & Aptroot lacking any lichen substances (Cáceres et al. 2013) and yet another from South Australia, i.e. *M. kartana* Kantvilas & Coppins containing gyrophoric acid (Kantvilas 2018). However, the phylogenetic relationships of these species are still uncertain due to the lack of molecular data. These studies also show that phenotypical variation within the *M. prasina* group may still be underestimated and requires further studies.

This study is based on specimens from years of collection in Belgium, France, Germany, Portugal (including Madeira and the Azores), Poland, Romania and the Netherlands. Using these collections for a phylogenetic reconstruction, six new species, belonging to the *M. prasina* group, are described by means of morphological, anatomical, chemical and molecular data. Moreover, by reconstructing ancestral states, the evolution of diagnostic traits, that are traditionally used for the taxonomic classification of species belonging to the *M. prasina* group, were investigated. Infra-group divisions and the role of secondary metabolites for species taxonomy within the *M. prasina* group were also evaluated. The production of selected secondary metabolites is further analysed (i.e. gyrophoric, methoxymicareic, micareic, prasinic and thiophanic acids), as well as the presence of several pigments in the apothecia commonly used in lichen taxonomy (Meyer and Printzen 2000) (i.e. Sedifolia-grey, Elachista-brown, Cinereorufa-green and Superba-brown). Ancestral state reconstruction of morphological characters i.e. goniocysts, isidia and soredia is also performed.

## Materials and methods

### Materials

Material of the new species, including samples used for DNA analyses, is deposited in KTC, UGDA and LG, with additional specimens stored in private herbaria of van den Boom and Brand.

### Morphology and chemistry

Apothecial sections and squashed thallus preparations were studied in tap water with or without the addition of C (commercial bleach) and K (water solution of potassium hydroxide) (Orange et al. 2001). Dimensions of all anatomical features were measured in water. Thin layer chromatography (TLC) was used for the determination of lichen substances according to the standard methods (Orange et al. 2001). All samples were studied in solvent C. The nomenclature of apothecial pigments follows Meyer and Printzen (2000). Crystalline granules were studied in polarised light (see Launis et al. 2019a, b).

### Taxon sampling for DNA

A total of 63 new sequences were generated for this study (Suppl. material 2, Table S1). Additional sequences of mtSSU, *Mcm7* and ITS rDNA from specimens of the *Micarea prasina* group were obtained from GenBank (Suppl. material 2, Table S1). Moreover, sequences of the above-mentioned markers from specimens of *M. adnata* Coppins, *M. elachista* (Körb.) Coppins & R. Sant., *M. globulosella* (Nyl.) Coppins, *M. misella*, *M. peliocarpa* (Anzi) Coppins & R. Sant., *M. pycnidiophora* Coppins & P. James, *M. stipitata* Coppins & P. James and *M. synotheoides* (Suppl. material 2, Table S1), which were shown to be outside the group (e.g. Launis et al. 2019a) were also obtained from GenBank. In total, sequences of 119 specimens were subjected to analyses. *Micarea peliocarpa* (Anzi) Coppins & R. Sant. was chosen as the outgroup, based on the study of Launis et al. (2019a).

### DNA extraction, PCR amplification and DNA sequencing

DNA was extracted directly from pieces of thalli using a modified CTAB method (Guzow-Krzemińska and Węgrzyn 2000). DNA extracts were used for PCR amplification and 25 μl of PCR mix contained 1U of Taq polymerase (Thermo Scientific) or 1U of DreamTaq polymerase (Thermo Scientific) and appropriate buffer, 0.2 mM of each of the four dNTPs, 0.5 μM of each primer and 10–50 ng of genomic DNA. PCR amplifications were performed using a Mastercycler (Eppendorf).

Amplifications of mtSSU rDNA, employing mrSSU1 and mrSSU3R primers (Zoller et al. 1999), were performed using the following conditions: initial denaturation at 95 °C for 10 min followed by 6 cycles at 95 °C for 1 min, 62 °C for 1 min and 72 °C for 105 s and then 30 cycles at 95 °C for 1 min, 56 °C for 1 min and 72 °C for 1 min, with a final extension step at 72 °C for 10 min.

Amplifications of the *Mcm7* region employing Mcm7_AL1r and Mcm7_AL2f primers (Launis et al. 2019a) were performed using the following conditions: initial denaturation at 94 °C for 5 min, followed by 38 cycles at 94 °C for 45 s (denaturation), 56 °C for 50 s (annealing) and 72 °C for 1 min (extension), with the final extension at 72 °C for 5 min.

Amplifications of the ITS region employed the following primer pairs: ITS1F (Gardes and Bruns 1993) and ITS4 (White et al. 1990) or ITS 5 and ITS4A (Kroken and Taylor 2001) or nu-SSU-1626-5’ (Gargas and DePriest 1996) and nu-LSU-136-3’ (Döring et al. 2000). The following PCR cycling parameters were applied to amplify nuclear ITS region: an initial denaturation at 94 °C for 3 min, followed by 35 cycles at 94 °C for 30 s, 54 °C for 30 s (for ITS1F and ITS4 or nu-SSU-1626-5’ and nu-LSU-136-3’ primers) or 62 °C for 30 s (for ITS5 and ITS4A primers) and 72 °C for 1 min, with a final extension at 72 °C for 7 min. PCR products were visualised on agarose gels in order to determine DNA fragment lengths. Subsequently, PCR products were purified using Clean-up Concentrator (A&A Biotechnology) following the manufacturer’s protocol or 10 μl of PCR products were treated with a mixture of 20 units of Exonuclease I and 2 units of FastAP Thermosensitive Alkaline Phosphatase enzymes (Thermo Scientific) to remove unincorporated primers and nucleotides. Treatment with those enzymes was carried out at 37 °C for 15 min, followed by incubation at 85 °C for 15 min to completely inactivate both enzymes. Sequencing of each PCR product was performed in Macrogen (www.macrogen.com) using the PCR primers.

### Sequence alignment and phylogenetic analysis

The newly generated sequences (GenBank accession numbers are given in Suppl. material 2, Table S1) were compared to the sequences available in the GenBank database (http://www.ncbi.nlm.nih.gov/BLAST/) using BLASTn search (Altschul et al. 1990) in order to confirm their identity. The sequences of each marker were aligned with sequences of selected representatives of the genus *Micarea* obtained from GenBank (list of specimens and GenBank Accession Numbers are given in Suppl. material 2, Table S1). Alignment was performed using Seaview software (Galtier et al. 1996; Gouy et al. 2010) employing the Muscle option, followed by manual optimisation. Portions of the alignment with ambiguous positions that might not have been homologous and terminal ends were excluded from the analyses. As the gene trees for each marker did not show any strongly supported conflicts, three datasets were combined into a concatenated matrix in the Seaview software (Galtier et al. 1996; Gouy et al. 2010) and the final alignment was deposited in Treebase (Accession No. S24731).

Partition Finder 2 (Lanfear et al. 2016), implemented at CIPRES Science Gateway (Miller et al. 2010), was used to determine the best substitution model for each partition under Akaike Information Criterion (AIC) and greedy search algorithm (Lanfear et al. 2012). The following models were found: TVM+I+G+X for mtSSU, TRN+I+G+X for *Mcm7* and GTR+I+G+X for ITS regions.

The data were analysed using a Bayesian approach (MCMC) in MrBayes 3.2 (Huelsenbeck and Ronquist 2001; Ronquist and Huelsenbeck 2003) and best models determined by Partition Finder 2 were employed. Two parallel runs were performed, each using four independent chains and 10 million generations, sampling trees every 1,000^th^ generation. Tracer v. 1.5 (Rambaut and Drummond 2007) was used to ensure that stationarity was reached by plotting the log-likelihood values of the sample points against generation time. Posterior probabilities (PP) were determined by calculating a majority-rule consensus tree generated from the 15,002 post-burn-in trees of the 20,002 trees sampled by the two MCMC runs, using the sumt option of MrBayes.

Maximum likelihood analyses were performed using RaxML HPC v.8 on XSEDE (Stamatakis 2014) under the GTRGAMMAI model on CIPRES Science Gateway (Miller et al. 2010). Rapid bootstrap analyses were performed with 1,000 bootstrap replicates (BS). The RAxML tree did not contradict the Bayesian tree topology for the strongly supported branches. Therefore, only the maximum likelihood tree is shown with the posterior probabilities (PP) of the Bayesian analysis and the bootstrap support values added near the internal branches. BS ≥ 70 and PP ≥ 0.95 were considered significant. Phylogenetic trees were visualised using FigTree v. 1.4.2, in which the clades for previously described taxa are collapsed (Rambaut 2012).

### Ancestral character state reconstruction

Morphological and chemical characters from taxa of the *Micarea prasina* group and selected outgroup taxa were obtained from herbarium material and complemented with data from literature. In order to reduce the number of missing data in our dataset, we did not include *M. pycnidiophora*, *M. stipitata* and *M. synotheoides*, which do not belong to the *M. prasina* group and for which mtSSU sequences were only available and *Micarea* sp. lineage A, which represents a single specimen that has not been formally described. The following secondary metabolites were analysed: gyrophoric, methoxymicareic, micareic, prasinic and thiophanic acids. The presence of apothecial pigmentation was also analysed and the following pigments were noted: Sedifolia-grey, Elachista-brown, Cinereorufa-green and Superba-brown. The presence of selected morphological characters was also analysed, i.e. goniocysts, isidia and soredia. The morphological and chemical characters were coded as a multistate data matrix (Suppl. material 2, Table S2) and a binary dataset (Suppl. material 2, Table S3) and subjected to ancestral character state reconstruction using the parsimony model with characters treated as unordered and the likelihood method (Mk1 model) in Mesquite v.3.5 (Maddison and Maddison 2018). Ancestral state reconstructions were based on the topology of the consensus tree obtained using Mr Bayes 3.2 (Huelsenbeck and Ronquist 2001; Ronquist and Huelsenbeck 2003)

## Results

**Figure 1.**
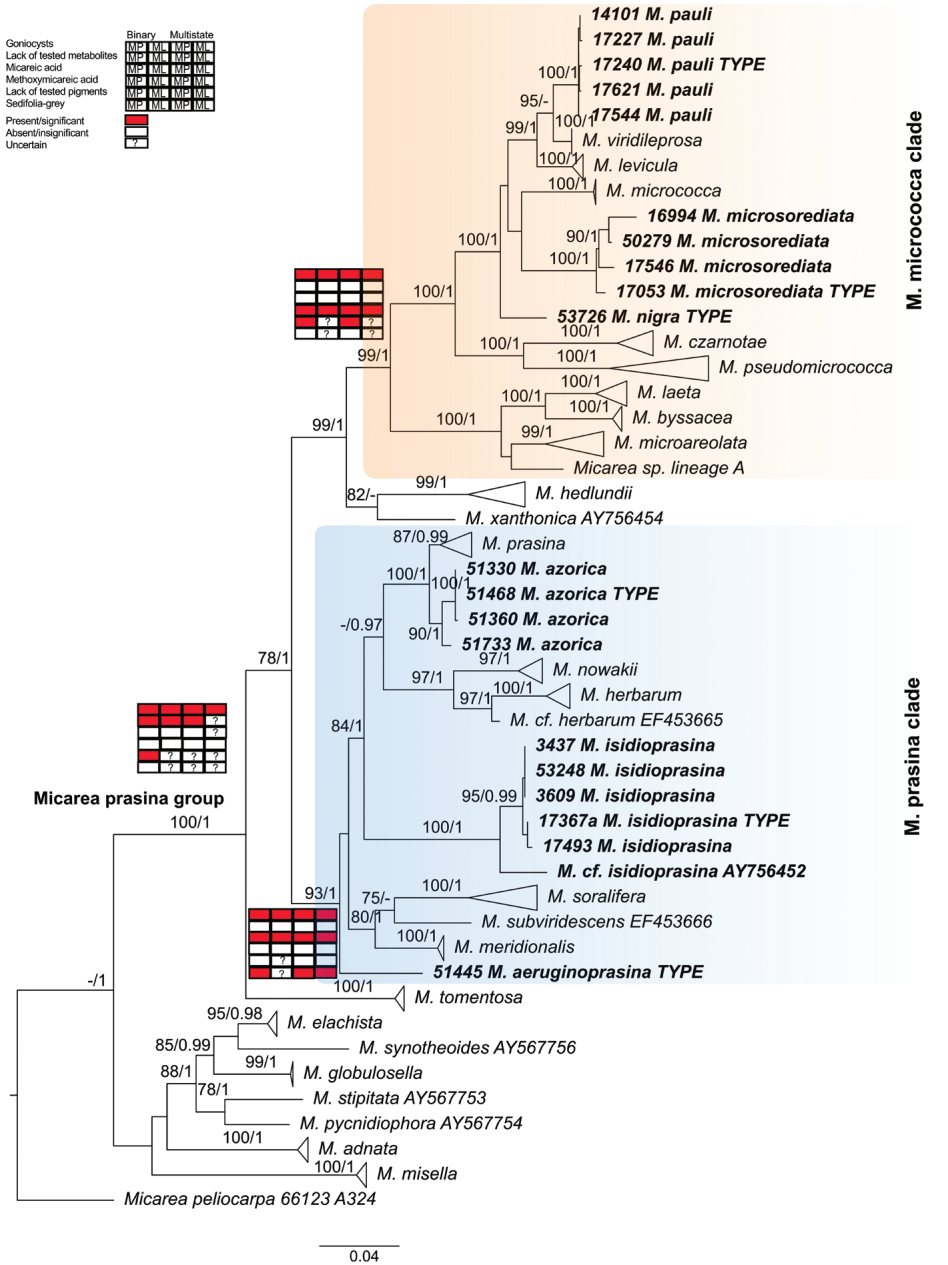
Maximum likelihood tree based on three-loci dataset. Bootstrap supports ≥ 70 for ML and posterior probabilities ≥ 0.95 (second value) for Bayesian methods are indicated near the branches. The highly supported clades with previously described species represented by numerous sequences are collapsed. Herbarium collection numbers for newly sequenced specimens precede the names of species and type specimens are marked. Newly described species are marked in **bold**. *Micarea micrococca* (orange) and *M. prasina* (blue) clades are indicated with shading. Ancestral states for selected characters reconstructed based on binary or multistate datasets using maximum parsimony (MP) or maximum likelihood methods (ML) are marked for the main clades: *M. prasina* group, *M. micrococca* clade and *M. prasina* clade using red (= present/significant) and white (= absent/insignificant) boxes or ? (= uncertain).

The final DNA alignment consisted of sequences obtained from 119 individual specimens and three markers, i.e. mtSSU, *Mcm7* and ITS rDNA, with a total of 1784 characters. Since the topologies from the maximum likelihood and Bayesian analyses did not show any strongly supported conflict, the maximum likelihood tree (RaxML Optimisation Likelihood was -14426.795913) is presented in Figure 1 with added posteriori probabilities from Bayesian analysis (Harmonic mean was -13101.16). In order to reduce the size of the tree, highly supported clades were collapsed for previously described taxa.

The phylogenetic reconstruction (Fig. 1) shows that the *M. prasina* group is highly supported and monophyletic (100 BS and 1 PP) and it agrees with previous phylogenies based on a mtSSU marker (e.g. Czarnota and Guzow-Krzemińska 2010; Guzow-Krzemińska et al. 2016) or three loci (Launis et al. 2019a). Two main lineages are further distinguished, i.e. the *M. micrococca* clade and the *M. prasina* clade with sequences of *M. tomentosa* forming a highly supported lineage, basal to the two clades (Fig. 1). Moreover, *M. hedlundii* and *M. xanthonica* are closely related (82 BS) and sister to the *M. micrococca* clade (Fig. 1).

The *Micarea micrococca* clade in Figure 1 (99 BS and 1 PP) consists mostly of species containing methoxymicareic acid. This group accommodates the newly described species *M. microsorediata*, *M. nigra* and *M. pauli*, as well as *M. byssacea*, *M. czarnotae*, *M. laeta*, *M. levicula*, *M. microareolata*, *M. micrococca*, *M. pseudomicrococca*, *M. viridileprosa* and an undescribed *Micarea* sp. (lineage A in Launis et al. 2019a). The closest relatives to *M. pauli* are *M. viridileprosa* and *M. levicula* (99 BS and 1 PP), while the relationships of *M. micrococca*, *M. microsorediata* and *M. nigra* remain unresolved.

The *Micarea prasina* clade (93 BS and 1 PP) consists mostly of species containing micareic acid and accommodates the newly described *M. aeruginoprasina*, *M. azorica* and *M. isidioprasina*, as well as *M. herbarum*, *M. meridionalis*, *M. nowakii*, *M. prasina*, *M. soralifera* and *M. subviridescens*. Several highly supported lineages are further distinguished within this clade. The newly described *M. azorica* forms a highly supported group with the type species of *M. prasina* s.str. (100 BS and 1 PP), whereas specimens of *M. prasina* form a well-supported group (87/0.99). Furthermore, they are sister to *M. nowakii* and *M. herbarum*, which are the only species within the *M. prasina* group developing almost entirely an endosubstratal thallus with only a few areoles. With the exception of *M. herbarum* and *M. nowakii*, this lineage (97 BS and 1 PP) also includes a sequence which seems to be different from both species (EF453665) and may indicate the existence of an undescribed taxon. Specimens of the newly described *M. isidioprasina* form a highly supported group (100 BS and 1 PP) with a single sequence from North America originally assigned to *M. prasina* (AY756452; see Andersen and Ekman 2005), but genetically more similar to *M. isidioprasina*. This sample is also morphologically similar to *M. isidioprasina* due to the isidioid thallus and pale apothecia (Czarnota and Guzow-Krzemińska 2010) and, therefore, is named here as M. cf. isidioprasina in Figure 1. *Micarea meridionalis*, *M. soralifera* and *M. subviridescens* form a highly supported group (80 BS and 1PP).

To investigate the diagnostic traits traditionally used for the taxonomic classification within the *M. prasina* group, we focused both on the *M. micrococca* and the *M. prasina* clades separately and the whole *M. prasina* together (Fig. 1) and employed both maximum parsimony and Mk1 models, based on the multistate and binary datasets (Suppl. material 2, Tables S2–S3 and Suppl. material 1, Figs S1–S15). The likelihoods for each set of characters are given in Suppl. material 2, Table S4. Our analyses found that the presence of methoxymicareic acid is restricted to the *M. micrococca* clade that accommodates several species containing this substance. However, *M. levicula* and *M. viridileprosa* are exceptions by producing gyrophoric acid (Suppl. material 1, Fig. S13). The ancestral state reconstructions show that the presence of methoxymicareic acid is the most parsimonious and the most likely ancestral state for the *M. micrococca* clade (Fig. 1, Tables 1–3 and Suppl. material 1, Figs S3, S13). On the other hand, micareic acid is the ancestral state for *M. prasina* clade in all analyses (Fig. 1, Tables 1–3, Suppl. material 1, Figs S2, S13). However, the reconstructions of ancestral state for the whole *M. prasina* group show the lack of any secondary metabolites in their ancestors in most of the analyses. However, the maximum likelihood analysis, based on the multistate dataset, suggests uncertainty as both the lack of any secondary metabolites and the presence of micareic acid are more likely than other states (Fig. 1, Tables 1–3 and Suppl. material 1, Figs S2, S13).

**Table 1.** Most parsimonious ancestral character states for selected subclades of the *M. prasina* group. The results that differ between maximum likelihood and maximum parsimony methods for each dataset are marked with *.

**Characters**	***M. prasina* group**	***M. micrococca* clade**	***M. prasina* clade**
Morphological characters	goniocysts	goniocysts	goniocysts
Secondary metabolites	lack of any substance*	methoxymicareic acid	micareic acid
Presence of apothecial pigments	uncertain (lack of pigments OR Sedifolia-grey)	lack of pigments or unknown*	Sedifolia-grey
Goniocysts	present	present	present
Isidia	absent	absent	absent
Soredia	absent	absent	absent
Gyrophoric acid	absent	absent	absent
Methoxymicareic acid	absent	present	absent
Micareic acid	absent	absent	present
Prasinic acid	absent	absent	absent
Thiophanic acid	absent	absent	absent
Cinereorufa-green	absent	absent	absent
Elachista-brown	absent	absent	absent
Sedifolia-grey	absent*	absent*	present*
Superba-brown	absent	absent	absent

The evolution of pigments, present in the apothecia, was also analysed, but some of the results remain uncertain in our analyses. Parsimony reconstructions, based on the binary dataset, suggest the lack of any pigment in the apothecia, while other analyses do not exclude the possibility that Sedifolia-grey pigment was present in the ancestor of *M. prasina* group (Fig. 1, Tables 1–3, Suppl. material 1, Figs S9–S12, S14). Moreover, the results obtained for the *M. micrococca* clade, using two different methods, are not fully consistent. Maximum parsimony analyses suggest a lack of pigments in their ancestors therefore resulting in multiple gains of Sedifolia-grey pigment and a single gain of Cinereorufa-green pigments in this lineage. However, maximum likelihood analyses show that both the lack of pigments in apothecia and the presence of Sedifolia-grey pigment may have occurred in their ancestor (Fig. 1, Tables 1–3, Suppl. material 1, Figs S9–S12, S14). In case of the *M. prasina* clade, maximum likelihood analyses, based on the binary dataset, give uncertain results as both presence and absence of Sedifolia-grey are equally likely; however parsimony analysis for the binary dataset and both analyses for the multistate dataset show the presence of Sedifolia-grey pigment in apothecia of their ancestor.

**Table 2.** Most likely ancestral character states in multistate analysis and their likelihoods for selected subclades of *M. prasina* group. Values for the most likely states are given in **bold**. The results that differ between maximum likelihood and maximum parsimony methods are marked with *

**Characters**	**State**	***M. prasina* group**	***M. micrococca* clade**	***M. prasina* clade**
Morphological characters	other or unknown	0.03672198	0.00009398	0.00056073
	goniocysts	**0.95494636**	**0.99972253**	**0.99844142**
	soredia	0.00411906	0.00009117	0.00008044
	isidia	0.00421261	0.00009232	0.00091742
Secondary metabolites	lack of any substances	**0.78962855**	0.05876201	0.00749498
	prasinic acid	0.01031084	0.00338938	0.00052042
	micareic acid	0.117972*	0.01320396	**0.98892236**
	methoxymicareic acid	0.04762568	**0.90867382**	0.00176536
	gyrophoric acid	0.01528514	0.00349062	0.00050881
	thiophanic acid	0.01917779	0.01248021	0.00078808
Presence of apothecial pigments	lack of pigment or unknown	**0.39914052**	**0.73149364**	0.03357191
	Sedifolia-grey	**0.44528926**	0.21519349*	**0.95640101**
	Cinereorufa-green	0.04803049	0.02251569	0.00293747
	Elachista-brown	0.05745307	0.01546335	0.00302814
	Superba-brown	0.05008665	0.01533383	0.00406148

Morphological characters, i.e. the presence of goniocysts observed in many species of the *M. prasina* group, soredia observed in *M. microsorediata*, *M. soralifera* and *M. viridileprosa* and isidia present in *M. aeruginoprasina*, *M. isidioprasina*, *M. nigra* and *M. pauli* were also evaluated (Fig. 1, Tables 1–3, Suppl. material 1 Figs S9–S12, S14). It was found that the presence of goniocysts is the most parsimonious and the most likely state for the ancestor of the *M. prasina* group in all analyses (Fig. 1, Tables 1–3 and Suppl. material 1, Figs S6, S15). However, this character has been lost in the lineage represented by *M. herbarum* and *M. nowakii* lacking goniocysts (Fig. 1, Tables 1–3 and Suppl. material 1, Figs S6, S15). Isidia and soredia evolved independently at multiple times in the *M. prasina* group resulting in the formation of isidiate thalli in the studied species, i.e. *M. aeruginoprasina*, *M. isidioprasina*, *M. nigra* and *M. pauli* or sorediate thalli in *M. microsorediata*, *M. soralifera* and *M. viridileprosa* (Fig. 1, Tables 1–3 and Suppl. material 1, Figs S7, S8, S15).

**Table 3.** Most likely ancestral character states based on analysis of binary dataset and their likelihoods for selected subclades of *M. prasina* group. Values for the most likely states are given in **bold**. The results that differ between maximum likelihood and maximum parsimony methods are marked with *

**Characters**	**State**	***M. prasina* group**	***M. micrococca* clade**	***M. prasina* clade**
Goniocysts	Present	**0.85180046**	**0.99078786**	**0.93297055**
	Absent	0.14819954	0.00921214	0.06702945
Isidia	Present	0.00354307	0.00079891	0.01513079
	Absent	**0.99645693**	**0.99920109**	**0.98486921**
Soredia	Present	0.00099039	0.00020816	0.00004518
	Absent	**0.99900961**	**0.99979184**	**0.99995482**
Gyrophoric acid	Present	0.00499412	0.00058456	0.00011577
	Absent	**0.99500588**	**0.99941544**	**0.99988423**
Methoxymicareic acid	Present	0.0023099	**0.85313833**	0.00010697
	Absent	**0.9986901**	0.14686167	**0.99989303**
Micareic acid	Present	0.02913989	0.00055219	**0.98044653**
	Absent	**0.97086011**	**0.99944781**	0.01955347
Prasinic acid	Present	0.00002438	0.0000051	0.00000103
	Absent	**0.99997562**	**0.9999949**	**0.99999897**
Thiophanic acid	Present	0.0000248	0.00000102	0.00001393
	Absent	**0.9999752**	**0.99999898**	**0.99998607**
Cinereorufa-green	Present	0.00002408	0.0000016	0.00000099
	Absent	**0.99997592**	**0.9999984**	**0.99999901**
Elachista-brown	Present	0.00002503	0.0000052	0.00000102
	Absent	**0.99997497**	**0.9999948**	**0.99999898**
Sedifolia-grey	Present	0.49999914*	0.49994168*	0.50492012
	Absent	0.50000086	0.50005832	0.49507988*
Superba-brown	Present	0.00050986	0.00004725	0.00000944
	Absent	**0.99949014**	**0.99995275**	**0.99999056**

## Discussion

Challenges in species delimitation within *M. prasina* group were already mentioned by Coppins (1983) and other authors (e.g. Czarnota 2007; Czarnota and Guzow-Krzemińska 2010; van den Boom et al. 2017; Launis et al. 2019a, b). Since Coppins (1983), who treated *M. prasina* in a wide sense with morphologically variable chemical races which were further recognised as distinct species, the introduction of molecular data revealed even greater variability within this group and numerous other species were described based on phenotypic and molecular data (e.g. Czarnota and Guzow-Krzemińska 2010; Guzow-Krzemińska et al. 2016; van den Boom et al. 2017; Launis et al. 2019a, b). Many species within this group have goniocystoid thallus, micareoid photobiont and Sedifolia-grey pigment in the apothecia, however a high variation in secondary metabolites production, which are treated as diagnostic characters, is observed within the *M. prasina* group. In the phylogenetic tree (Fig. 1) two main clades were distinguished; *M. micrococca* clade which groups mainly taxa containing methoxymicareic acid and *M. prasina* clade which mainly comprises species containing micareic acid. However, there are some exceptions as other substances may be produced by selected representatives of the group, e.g. gyrophoric, prasinic or thiophanic acids or some taxa do not produce any secondary metabolites. Within this group, numerous phenotypic differences are applied to distinguish species, e.g. size and shape of apothecia, size and type of paraphyses, size of ascospores, thallus structure including the vegetative diaspores and presence of pigments. Recently introduced crystalline granules showed to be valuable traits in the taxonomy of the group (Launis et al. 2019a, b). However, the application of molecular data seems to be essential to support delimitation of species within this group (e.g. Launis et al. 2019a, b; this study).

The evolution of new morphological characters involves multiple subsequent evolutionary steps. In our study, ancestral state reconstructions showed that the presence of goniocysts is the most parsimonious and most likely state for the ancestor of the *M. prasina* group (Fig. 1, Tables 1–3 and Suppl. material 1, Figs S6, S15). However, the development of goniocysts was apparently lost in some lineages during evolution as several species within the group do not develop such structures but produce other vegetative diaspores (soredia and/or isidia). Whether the structures from which soredia and isidia develop are goniocysts or areoles is not easy to assign. Based on literature, goniocysts are more or less round vegetative diaspores (therefore similar to soredia) and are produced from the endosubstratal parts of thalli multiple times to form a layer as in *M. prasina* s.str. (Coppins 1983; Barton and Lendemer 2014). As the thallus parts developing isidioid or soredioid diaspores did not resemble goniocysts as defined in previous works, we determined all these structures as areoles, as already proposed by Guzow-Krzemińska et al. (2016). Although soredia in the newly described *M. microsorediata* and recently recognised *M. soralifera* (Guzow-Krzemińska et al. 2016) may resemble goniocysts, they are at least at the beginning produced in delimited soralia over the thallus and differ in the structure and colour from the non-sorediate parts of thalli.

In our study, ancestral state reconstructions suggest that isidia evolved independently multiple times in this group of lichens resulting in the formation of almost entirely isidiate thalli in four species, i.e. *M. aeruginoprasina*, *M. isidioprasina*, *M. nigra* and *M. pauli* (Suppl. material 1, Figs S6–S8, S15). Prieto et al. (2013) suggested that losing an existing character could be expected to occur much more rapidly and in fewer steps than gaining a new character. A similar case is represented by sorediate species and the production of soredia developed in unrelated lineages. Only one lineage lost the ability to produce goniocysts or any other lichenised vegetative diaspores (i.e. *M. herbarum* and *M. nowakii*). Species belonging to this clade develop thin episubstratal thalli with few areoles or merely an endosubstratal layer (Czarnota 2007; van den Boom et al. 2017). The acquisition of different thallus organisation may have resulted from adaptation to drier ecological niches. Many collections of the species from this clade were found in drier and open habitats (Czarnota 2007; van den Boom et al. 2017). In comparison, taxa developing distinct episubstratal thalli seem to be confined to more humid and shaded localities (Czarnota 2007). However, this hypothesis needs further ecological studies.

Secondary metabolites have been extensively used in the chemotaxonomy of lichens. The *Micarea prasina* group shows a high variation in chemistry even in closely related species (e.g. Czarnota 2007; Czarnota and Guzow-Krzemińska 2010). Species belonging to this group produce gyrophoric, micareic, methoxymicareic and prasinic acids, as well as xanthones (Elix et al. 1984; Coppins and Tønsberg 2001; van den Boom and Coppins 2001). Gyrophoric acid is the simplest tridepside comprising three orsellinic units which originate from condensation of one acetyl-CoA and three malonyl-CoA units as shown by Mosbach (1964). Although gyrophoric acid is commonly produced in the genus *Micarea* (e.g. Coppins 1983; Czarnota 2007), in the *M. prasina* group, it is only present in *M. levicula* and *M. viridileprosa* and the still unsequenced *M. kartana* (Kantvilas 2018). Both *M. levicula* and *M. viridileprosa* belong to the *M. micrococca* clade which is otherwise characterised by the production of methoxymicareic acid.

Micareic and methoxymicareic acids are the most common secondary metabolites produced by species of the *M. prasina* group. They are structurally related diphenyl ethers (‘pseudodepsidones’) (Huneck and Yoshimura 1996), but they have a distincly different substitution pattern and probably also biosynthetic origin (Elix et al. 1984). As numerous diphenyl ethers co-occur with structurally related depsidones, it was hypothesised that they are biosynthesis precursors or catabolites of similarly substituted depsidones (Huneck and Yoshimura 1996). In the work on the secondary metabolites of chemical races of the *M. prasina* s.l., Elix et al. (1984) suggested that enzymatically induced Smiles rearrangement of *para*-depside prasinic acid might lead to the formation of micareic acid, a very likely biosynthetic pathway for this metabolite. They also pointed out that other rearrangements, such as nuclear hydroxylation followed by O-methylation, are necessary for the formation of methoxymicareic acid, but the actual order of those processes remain unknown. However, the chemical races of *M. prasina* s.l. they studied actually represent several species which were later distinguished as *M. micrococca* (methoxymicareic acid chemotype), *M. prasina* s.str. (micareic acid chemotype) and *M. subviridescens* (prasinic acid chemotype) (Coppins 2009); furthermore, other new species have also been recognised within the *M. prasina* group. Both micareic and methoxymicareic acids are produced by several species within the *M. prasina* group, while prasinic acid has only been reported from *M. subviridescens*. So far, no co-occurrence of any of those substances has been observed in any species within the *M. prasina* group.

Reconstructions of the ancestral state for the whole *M. prasina* group suggest that the most recent common ancestor did not produce any secondary metabolites. This may suggest that the production of a wide range of secondary metabolites in this group of lichens could have resulted from independent gains of ability to biosynthesise various substances during evolution. The scenario, in which the ability to produce micareic acid in the ancestor of *M. prasina* clade or methoxymicareic acid in the ancestor of *M. micrococca* clade being gained only once during evolution, seems to be reasonable since losing an existing character could be expected to occur more rapidly and in fewer steps than gaining a new character (e.g. Prieto et al. 2013). Those evolutionary events could have been followed with the loss of those traits in some lineages and successive independent gains of ability to biosynthesise prasinic (*M. subviridescens*) or gyrophoric acids (*M. levicula* and *M. viridileprosa*) in some species.

To summarise, our study showed that phenotypical variation within the *Micarea prasina* group has been previously underestimated and, based on field work and laboratory studies, six new species within this group are described (see Taxonomy).

## Taxonomy

### Micarea aeruginoprasina

Taxon classificationFungiLecanoralesPilocarpaceae

van den Boom, Guzow-Krzemińska, Brand & Sérus. sp. nov.

2476cb32-c0a2-580e-8d15-2bde67caa2b3

MycoBank No.: MB 831821

Fig. 2A

#### Diagnosis.

Species characterised by inconspicuous, pale brownish to moderately brownish, isidiate thallus, branched to coralloid isidia, emarginate, adnate to slightly convex apothecia measuring 0.1–0.5 mm in diam., which are pale cream to pale brown or aeruginose with pigment (Sedifolia-grey, K+ violet, C+ violet) present in hypothecium, (0–)1-septate ascospores measuring 9–14 × 4.5–5.5 µm and the production of micareic acid.

#### Type.

Portugal. Azores, Terceira, NW of Angra do Heroismo, W of Pico Gordo, Mistério dos Negros (N), trail from Lagoa do Negro to the West, 550 m alt., 38°44.15'N, 27°16.30'W, ± damp *Juniperus brevifolia* forest, with some young *Vaccinium cylindraceum*, on *Juniperus brevifolia*, 28 June 2014, P. & B. van den Boom 51445 (holotype LG; isotypes UGDA, hb v.d. Boom, mtSSU GenBank accession number: MK562024, *Mcm7* GenBank accession number: MN105888).

#### Description.

Thallus indeterminate, inconspicuous, thin, endosubstratal to episubstratal in non-isidiate parts as a thin film over the substrate or minutely granular, pale to moderately brown, isidiate; prothallus not visible; granules vertically proliferating to form isidia; isidia branched to coralloid, crowded or separated, up to 250 μm tall and 25 μm wide, with a distinct and complete hyphal layer; apothecia abundant, adnate to slightly convex, emarginate, rounded to slightly irregular, pale cream to pale brown or aeruginose, often different colours in a single apothecium, 0.1–0.5 mm in diam.; excipulum sometimes paler, instinct; hymenium 40–50 µm high, hyaline; hypothecium hyaline to pale aeruginose brownish (Sedifolia-grey), K+ violet, C+ violet; paraphyses, sparse, branched, 1.0–1.2(–1.5) µm wide, tips not widened and not pigmented; asci cylindrical to clavate, 35–40 × 11–14 µm, 8–spored; ascospores ellipsoidal to ovoid, (0‒)1-septate, 9–14 × 4.5–5.5 µm; pycnidia not observed; crystalline granules (studied in polarised light) visible in hypothecium and in thallus, soluble in K.

Photobiont micareoid, cells thin walled, 6–9 µm in diam., clustered in compact groups.

#### Chemistry.

Micareic acid detected by TLC. Sedifolia-grey in apothecia (hypothecium), its presence sometimes indistinct.

#### Habitat and distribution.

In the type locality *Micarea aeruginoprasina* grows abundantly on trunks of *Juniperus brevifolia*, in a subnatural degradated forest, dominated by *J. brevifolia* shrubs and trees. In other localities, it was found on *Cryptomeria* and *Erica* trunks, also in forested areas.

The new species is only known from the island Terceira in the Azores, where it is known from several localities.

#### Etymology.

The epithet refers to the often aeruginose colour of the apothecia and the resemblance in secondary chemistry to *M. prasina*.

#### Additional specimens examined.

Portugal. Azores, Terceira, NW of Angra do Heroismo, south edge of Reserva Florestal da Lagoa das Patas, area around a pond ‘Lagoa das Patas’, mature *Cryptomeria* trees and some *Camellia* shrubs, on *Cryptomeria*, 38°43.01'N, 27°17.32'W, 520 m alt., 28 June 2014, P. & B. van den Boom 51878 (hb v.d. Boom); NW of Angra do Heroismo, NNE of Santa Bárbara, Serra de Santa Bárbara, road to the summit, forests with mainly *Cryptomeria* trees, trees at edge of forest, on *Cryptomeria*, 38°43.49'N, 27°19.33'W, 800 m alt., 1 July 2014, P. & B. van den Boom 51622 (hb v.d. Boom); NE of Serreta, north trail to Lagoínha, forest with *Cryptomeria japonica*, *Myrica faya*, *Erica*, etc., on *Erica*, 38°45.28'N, 27°20.50'W, 500 m alt., 2 July 2014, P. & B. van den Boom 51691 (hb v.d. Boom).

#### Notes.

This species is unique within the group due to the presence of the Sedifolia-grey pigment in hypothecium. It is similar to *M. prasina* because of its production of micareic acid, but the latter has Sedifolia-grey pigment in the epihymenium and its thallus consists of goniocysts (isidiate in *M. aeruginoprasina*). However, it is not closely related to *M. prasina*, being resolved as basal in the *M. prasina* clade and the sequences of their molecular markers are very different. In the Azores archipelago, the most widespread *prasina*-like species is *M. azorica*, newly described in this paper, which, however, is not isidiate and contains Superba-brown in the apothecia. *Micarea aeruginoprasina* resembles *M. byssacea*, which can have somewhat the same coloured and adnate apothecia; however, *M. byssacea* is not isidiate, contains methoxymicareic acid and the apothecial pigment is absent in hypothecium (Czarnota and Guzow-Krzemińska 2010). Morphologically, the new species is similar to *M. levicula*, especially due to the finely isidiose thallus and the adnate apothecia, which are, however, paler in *M. levicula* and that species contains gyrophoric acid (Coppins 1983; Brand et al. 2014).

*Micarea isidioprasina*, *M. nigra* and *M. pauli* also have isidiate thalli, but only *M. aeruginoprasina* has pale cream to pale brown or aeruginose apothecia. *Micarea isidioprasina* and *M. pauli* are often sterile and, to date, *M. aeruginoprasina* and *M. nigra* have always been found with apothecia, but, based only on the thallus characters, *M. nigra* and *M. pauli* can be distinguished due to the production of methoxymicareic acid and *M. isidioprasina* has green isidia (shades of brown in *M. aeruginoprasina*).

**Figure 2.**
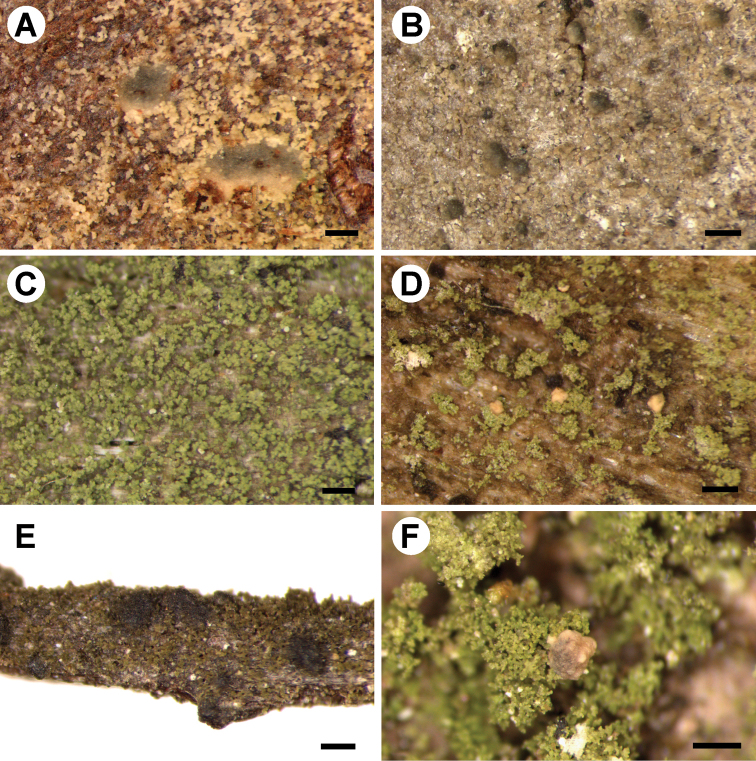
Morphology of newly described *Micarea* species **A***M. aeruginoprasina* (holotype) **B***M. azorica* (holotype) **C***M. isidioprasina* (holotype) **D***M. microsorediata* (holotype) **E***M. nigra* (holotype) **F***M. pauli* (holotype). Scale bars: 200 µm (**A−C, E**); 300 µm (**D, F**).

### Micarea azorica

Taxon classificationFungiLecanoralesPilocarpaceae

van den Boom, Guzow-Krzemińska, Brand & Sérus. sp. nov.

da6b9548-7082-56a4-b949-a21dbdaae760

MycoBank No.: MB 831822

Fig. 2B

#### Diagnosis.

Species characterised by pale to moderately brownish thallus consisting of goniocysts, convex to subglobose, emarginate, pale greyish-brown to dark brown (with Superba-brown pigment) apothecia measuring 0.1–0.3 mm in diam., (0–)1-septate, narrowly ellipsoidal to ovoid ascospores measuring 9–11 × (2.5–)3–4 µm, sessile to slightly stalked, pale to moderately brown mesopycnidia, bacillar mesoconidia measuring 6.5–8 × 0.9–1.1 µm and the production of micareic acid.

#### Type.

Portugal. Azores, Terceira, NW of Angra do Heroismo, south edge of Reserva Florestal da Lagoa das Patas, area around a pond ‘Lagoa das Patas’, 520 m alt., 38°43.01'N, 27°17.32'W, mature *Cryptomeria* trees and *Camellia* shrubs, on *Cryptomeria japonica*, 28 June 2014, P. & B. van den Boom 51468 (holotype LG; isotypes UGDA, hb v.d.Boom, mtSSU GenBank accession number: MK562026, *Mcm7* GenBank accession number: MN105891).

#### Description.

Thallus inconspicuous, thinly scurfy to somewhat farinose-granular, pale to moderately brownish and consisting of goniocysts; prothallus not seen; apothecia abundant, convex to subglobose, emarginate, pale greyish-brown to dark brown, often unevenly coloured in a single apothecium (partly dark, partly pale), 0.1‒0.3 mm in diam.; hymenium ca. 32‒40 µm tall; epihymenium with grey-brown pigment, K‒, C‒ (Superba-brown); hypothecium hyaline; paraphyses, abundant, branched, ca. 1.0–1.5(–1.8) µm wide, tips not widened and not pigmented; asci 25–35 × 11–14 µm, 8-spored; ascospores narrowly ellipsoidal to ovoid, (0–)1-septate, 9–11 × (2.5–)3–4 µm; mesopycnidia occasionally abundant, sessile to slightly stalked, 40–60 µm in diam., pale to moderately brown, the ostiole sometimes gaping; mesoconidia bacillar, simple, 6.5‒8 × 0.9‒1.1 µm; crystalline granules (studied in polarised light) visible in epithecium and in thallus, soluble in K.

Photobiont micareoid, cells thin-walled, 4–10 µm in diam., clustered in compact groups.

#### Chemistry.

Micareic acid detected by TLC. Superba-brown in apothecia (epihymenium).

#### Habitat and distribution.

To date, known only from the Azores archipelago (Terceira island) from three localities where it was found on bark of trees.

#### Etymology.

The name refers to the archipelago of the Azores, where the species occurs.

#### Additional specimens examined.

Portugal. Azores, Terceira, NW of Angra do Heroismo, Reserva Florestal Viveira da Falca, 460 m alt., 38°42.90'N, 27°16.78'W, picnic area with many mature *Cryptomeria* trees, some *Acer* trees and *Camellia*, on *Cryptomeria*, 28 June 2014, P. & B. van den Boom 51330 (hb. v.d. Boom); N of Serreta, Reserva Florestal da Serreta, 80 m alt., 38°46.27'N, 27°21.42'W, picnic area in open forest with mixed trees and shrubs, on tree, 2 July 2014, P. & B. van den Boom 51733 (hb. v.d. Boom).

**Notes.** The new species is resolved as sister to *M. prasina* s.str. with strong support, being morphologically and chemically similar to that species, but differing in the absence of the Sedifolia-grey pigment, responsible for the typical reaction K+ violet in *M. prasina* s.str (Coppins 1983; Czarnota 2007; Launis et al. 2019a). Instead of Sedifolia-grey pigment, Superba-brown is present in *M. azorica*.

The identity of *M. prasina* s.str. has been recently solved by Launis et al. (2019a, b) and its occurrence is confirmed from boreal and temperate Europe (Finland, Germany, Poland) and Eastern North America (Canada: New Brunswick and USA: Maine) (Launis et al. 2019b; this paper). Other records need confirmation as, previously, other species have been included in the variation of *M. prasina*.

*Micarea azorica* resembles *M. lithinella* (Nyl.) Hedl. due to its brownish, convex to subglobose small apothecia, but the latter is mainly a saxicolous species, has smaller conidia, 4‒5.5 × 0.5‒1 µm and does not contain secondary metabolites (Coppins 1983; Czarnota 2007).

### Micarea isidioprasina

Taxon classificationFungiLecanoralesPilocarpaceae

Brand, van den Boom, Guzow-Krzemińska, Sérus. & Kukwa sp. nov.

077652a9-8a0d-57b5-885b-a14d0c29b79e

MycoBank No.: MB 831823

Fig. 2C

#### Diagnosis.

Species characterised by granular-isidiate thallus, pale grey to grey-beige apothecia, 0–1-septate, ovoid, ellipsoidal or oblong ascospores measuring 7–13 × 3.5–4.5 μm and the presence of micareic acid.

#### Type.

POLAND. Równina Bielska, Białowieża Primeval Forest, Białowieża National Park, forest section no 256, *Pino-Quercetum*, on wood of log, 21 Aug 2015, M. Kukwa 17367a, A. Łubek (holotype UGDA; isotype KTC, ITS GenBank accession number: MN095789, mtSSU GenBank accession number: MK562016, *Mcm7* GenBank accession number: MN105897).

#### Description.

Thallus crustose, granular-isidiate, indeterminate, endosubstratal to rarely episubstratal in non-isidiate parts and then as a thin greenish film over the substrate or minutely areolate, isidiate; prothallus not seen; areoles up to 0.05 mm in diam., green, soon developing isidia; isidia abundantly branched and coralloid, crowded and forming an almost continuous layer locally over the substrate, but in younger parts of thalli separated, green to olive green (Sedifolia-grey, K+ violet), up to 250 μm tall and 25 μm wide, with a distinct and complete hyphal layer; apothecia rarely developed, white to beige, some patchily grey, up to 0.45 mm in diam., convex; excipulum poorly developed, as a narrow, hyaline zone, hyphae radiating, branched and anastomosing; hymenium up to 50 μm tall, hyaline; epihymenium and hypothecium hyaline; paraphyses of one type, 1–1.5 μm thick, sparse, mostly apically branched and anastomosed, hyaline throughout; asci cylindrical-clavate, 30–45 × 12–15 μm, 8-spored; ascospores, 0–1-septate, ovoid, ellipsoidal or oblong, 11–14 × 3.5–4.5 μm; pycnidia not seen; crystalline granules (studied in polarised light) present rather sparsely in hymenium (as strands between asci and paraphyses) and abundantly in isidia, soluble in K.

Photobiont chlorococcoid, micareoid, cells globose to ellipsoidal, 4–7 μm in diam.

#### Chemistry.

Micareic acid detected by TLC. Sedifolia-grey pigment present in outermost parts of some isidia.

#### Habitat and distribution.

The species grows on wood (decomposing logs) and acidic bark of trees in various forest communities in well preserved forest.

To date, it is known from Belgium, Germany, France, Poland and Romania.

#### Etymology.

The name of the new species refers to the presence of isidia and the chemistry of *M. prasina*.

#### Additional specimens examined.

Belgium. Herbeumont, forest by the Semois river, 265 m alt., 49°45'N, 05°13'E, on *Quercus* tree in forest, 2013, E. Sérusiaux 3609 (LG). France. Vosges, Dépt. Haut-Rhin, Hohneck, Frankenthal nature preserve, 48°02'N, 07°01'E, 1100 m alt., on dead *Fagus* in forest, 2013, E. Sérusiaux LG DNA 3437 (LG). Germany. Niedersachsen, S of Goslar, Rammelsberg, 360 m alt., 51°53.01'N, 10°25.23'E, trail along *Picea* forest and brooklet with *Acer*, *Alnus* and *Betula* trees, 12 May 2015, P. & B. van den Boom 53248 (hb. v.d. Boom). Poland. Roztocze Środkowe, Roztoczański National Park, S of Zwierzyniec village, Bukowa Góra nature reserve, 50°35'47"N, 22°57'48"E, ca. 280 m alt., beech forest, on wood of log, 15 Sept 2015, M. Kukwa 17493 (UGDA); Równina Bielska, Białowieża Primeval Forest, Białowieża National Park, forest section no 256, *Carici elongatae-Alnetum*, on wood of logs, bark *Picea abies* and *Alnus glutinosa*, Aug 2014, M. Kukwa 14030, 14038, 14107, 14112, A. Łubek (KTC, UGDA); ibidem, *Circaeo*-*Alnetum*, on wood of log, Aug 2014, M. Kukwa 13299, A. Łubek (KTC, UGDA); ibidem, *Tilio*-*Carpinetum*, on wood of log, Aug & Oct 2014, M. Kukwa 13418, 14358, A. Łubek (KTC, UGDA); ibidem, *Circaeo-Alnetum*, on wood of snag, Oct 2014, M. Kukwa 14243, A. Łubek (KTC, UGDA). Romania. W of Brasov, S of Zarnesti, Praia Craiului National Park, 1350 m alt., 45°31'N, 25°16'E, on *Fagus* inside forest, 2016, E. Sérusiaux LG DNA 6260 & 6265 (LG).

#### Notes.

*Micarea isidioprasina* is an isidiate species of the *M. prasina* group containing micareic acid as the main secondary metabolite. It is usually sterile and in Poland often grows in similar habitats with *M. pauli*, a species described in this paper, from which it can be separated with certainty by analyses of secondary metabolites, as the latter contains methoxymicareic acid.

*Micarea aeruginoprasina* and *M. nigra* also develop similar isidiate thalli, but *M. aeruginoprasina* has pale cream to pale brown or aeruginose apothecia (often mottled with all colours in the same apothecium) and *M. nigra* develops dark greyish to black apothecia. When sterile, all three species may be more difficult to separate, especially *M. aeruginoprasina* which also produces micareic acid (*M. nigra* contains methoxymicareic acid), but that species has pale brown isidia. Additionally, the so far known distributions of all three species do not overlap and *M. aeruginoprasina* and *M. nigra* are known from the Azores and continental Portugal, respectively.

Micareic acid is also the main secondary metabolite in the somewhat morphologically similar *M. prasina*, but the latter is not isidiate, often richly fertile and its thallus consists of goniocysts (Czarnota 2007; Launis et al. 2019a, b).

### Micarea microsorediata

Taxon classificationFungiLecanoralesPilocarpaceae

Brand, van den Boom, Guzow-Krzemińska, Sérus. & Kukwa sp. nov.

71e880c7-0f2a-5261-be42-e7560d6b29be

MycoBank No. MB 831824

Fig. 2D

#### Diagnosis.

Species morphologically similar to *Micarea viridileprosa*, characterised by sorediate thallus, delimited or diffuse and confluent soralia with green or locally bluish soredia produced from the thallus areoles, white and immarginate when mature apothecia, 0.2–0.3 mm in diam., cylindrical to ellipsoidal (0–)1-septate ascospores measuring 9.5–13 × 2.8–3.5 µm and the presence of methoxymicareic acid.

#### Type.

Poland. Wysoczyzna Żarnowiecka, Pużyckie Łęgi nature reserve, 54°38'N 17°51'E, *Circaeo*-*Alnetum*, on wood of log, 12 Aug 2015, M. Kukwa 17053 (holotype UGDA, ITS GenBank accession number: MN095791, mtSSU GenBank accession number: MK562012, *Mcm7* GenBank accession number: MN105906).

#### Description.

Thallus diffuse, up to 10 cm wide, consisting of finely granular soredia, often with a powdery appearance, vivid green or green, sometimes with bluish tinge; prothallus not seen; areoles up to 25 µm in diam., green, soon bursting to produce soredia; soralia at first delimited, produced from small, convex areoles, soon fused and confluent, sometimes forming a sorediate continuous layer; soredia simple, up to 20 µm in diam., sometimes slightly elongated or in more or less rounded consoredia up 35 µm in diam. apothecia rarely present, adnate, first with indistinct margin, then immarginate, 0.2–0.3 mm in diam., white or slightly brownish; excipulum in young apothecia present, 15–25 µm wide, of thin irregular hyphae; hymenium ca. 30‒42 µm tall; epihymenium and hypothecium hyaline; paraphyses thick (in K), branched and anastomosing, ca. 1.2‒1.5 µm wide; asci 29–35 × 7–10 µm, 8-spored; ascospores cylindrical to ellipsoidal, 9.5–13 × 2.8–3.5 µm, (0–)1-septate; micropycnidia present in some specimens, ca. 60 µm in diam., with dark brown tops (K–); microconidia narrow fusiform to bacilliform, 7 × 0.8 µm; mesopycnidia, mesoconidia 3.8 × 1.4 µm; crystalline granules (studied in polarised light) visible in hymenium and in thallus, soluble in K.

Photobiont micareoid, cells thin-walled, 4–8(–9) µm in diam.

#### Chemistry.

Methoxymicareic acid detected by TLC. Soredia in exposed habitats with Sedifolia-grey pigment, K+ violet.

#### Habitat and distribution.

The new species occurs on acidic bark of various trees such as *Alnus*, *Betula*, *Fagus* and *Quercus*, usually in humid forests, also on decaying wood (logs and stumps) and rarely on terrestrial decaying mosses in, for example, steep slopes in heath and dunes. It is a very common species in the south of the Netherlands and some areas in Poland and is mostly found on microhabitats where only few other lichens species co-occur. On several occasions, *Normandina pulchella* (Borrer) Nyl. and squamules of *Cladonia* spp. are the only accompanying lichens.

To date, the species has been found in Belgium, Germany, the Netherlands, Poland and Portugal.

One specimen of *Micarea microsorediata* was invaded by *Nectriopsis micareae* Diederich, van den Boom & Ernst (see below additional specimens examined).

#### Etymology.

The epithet refers to the production of soredia and the similarity to *M. micrococca* due to the same secondary chemistry.

#### Additional specimens examined.

Belgium. Limburg, N of Achel, Rozendaal, 51°17.0'N, 5°29.9'E, 35 m alt., *Pinus* forest with *Betula* and *Quercus* trees, on wood of fallen decaying trunk, 28 Dec. 2018, P. & B. van den Boom 58046 (hb v.d. Boom); NE of Achel, near Tomp, 51°16.10'N, 5°29.8'E, 35 m alt., along small road, *Pinus* forest with *Betula* and *Quercus* trees, on *Betula*, 28 Dec. 2018, P. & B. van den Boom 58052 (hb v.d. Boom); NE of Lommel, Kolonie, E of ‘Afwateringskanaal’, 51°14.40'N, 5°23.6'E, 50 m alt., *Pinus* forest, on *Prunus*, 28 Dec. 2018, P. & B. van den Boom 58054 (hb v.d. Boom); ENE of Lommel, E of Kolonie, 51°15.50'N, 5°24.35'E, 40 m alt., between edge of *Pinus* forest and edge of reserve Hageven, on *Betula* and *Quercus robur*, 28 Dec. 2018, P. & B. van den Boom 58055, 58056 (hb v.d. Boom). Germany. Nedersaksen, N of Bentheim, NE of Wengsel, Isterberg, 52°21.4'N, 7°9.0'E, *Pinus* forest with some sandstone outcrops, on *Quercus rubra*, 26 Aug. 2015, M. & D. Brand, P. & B. van den Boom 53633 (hb v.d. Boom). Poland. Bory Tucholskie, Kręgi Kamienne nature reserve, close to Wda river, NW from Odry, 53°53'59.50"N, 17°59'42.07"E, black alders close to river, on *Alnus glutinosa*, 19 June 2018, M. Kukwa 19991 (UGDA); Równina Bielska, Białowieża Forest, Białowieża National Park, forest section no 256, *Peucedano*-*Pinetum*, on twig of *Picea abies*, *Quercus robur* and wood of log, May 2014, M. Kukwa 13462, 13660, 13675, 13778, A. Łubek (KTC); ibidem, *Pino*-*Quercetum*, on *Quercus robur*, Aug. 2014, M. Kukwa, 13321, A. Łubek (KTC, UGDA); ibidem, *Tilio-Carpinetum*, on *Quercus robur*, Aug. 2014, M. Kukwa 13420a, 13392 (as admixture in a specimen of *Biatora ligni-mollis* T. Sprib. & Printzen), A. Łubek (KTC, UGDA); ibidem, *Carici elongatae*-*Alnetum*, on bark of log, *Alnus glutinosa* and *Picea abies*, Aug. 2014, M. Kukwa, 14000, 14002, 14012, 14045, 14109, A. Łubek (KTC, UGDA); ibidem, *Circaeo*-*Alnetum*, on wood of snag, Oct. 2014, M. Kukwa, 14139, A. Łubek (KTC); ibidem, *Tilio*-*Carpinetum*, on wood of branch of the log, Oct. 2014, M. Kukwa, 14350, A. Łubek (KTC, UGDA); ibidem, *Peucedano*-*Pinetum*, on *Alnus glutinosa*, March 2015, M. Kukwa, 13308, A. Łubek (KTC, UGDA); ibidem, *Carici elongatae*-*Alnetum*, on wood of stump, May 2015, M. Kukwa, 15789, A. Łubek (KTC, UGDA); ibidem, *Circaeo-Alnetum*, on *Alnus glutinosa*, 29 Sept. 2015, M. Kukwa, 17546, A Łubek (KTC, UGDA); ibidem, *Querco*-*Piceetum*, on *Carpinus betulus*, Aug 2015, M. Kukwa, 17379, 17436, A. Łubek (UGDA; KTC); ibidem, *Circaeo*-*Alnetum*, on *Alnus glutinosa*, 1 Oct. 2015, M. Kukwa, 17592, A Łubek (KTC, UGDA); ibidem, *Pino*-*Quercetum*, on wood of log, 4 Oct. 2015, M. Kukwa, 17641, A. Łubek (KTC, UGDA); Wybrzeże Słowińskie, Białogóra nature reserve, forest section no. 14, 54°49'29"N, 17°57'21"E, *Empetro nigri*-*Pinetum*, on *Pinus sylvestris*, 21 April 2010, M. Kukwa 7721 (UGDA); ibidem, forest section no. 22, 54°49'27"N, 17°57'52"E, open area with few trees and *Myrica gale*, on *Betula pendula*, 22 Sept. 2010, M. Kukwa, 8280, A. Jabłońska, M. Oset (UGDA); Wysoczyzna Polanowska, Skotawskie Łąki nature reserve, 54°16'03"N, 17°33'37"E, large group of black alders, on *Alnus glutinosa*, 11 April 2017, M. Kukwa, 19212, 19219 (UGDA); ibidem, by NE part of Lipieniec lake, 54°15'44"N, 17°33'26"E, group of black alder trees on meadow, on *Alnus glutinosa*, 27 June 2017, M. Kukwa, 19800a, 19801, 19806 (UGDA); ibidem, by unnamed lake (N of Lipieniec lake), 54°15'46"N, 17°33'03"E, black alder forest, by lake, on *Alnus glutinosa*, *Pinus sylvestris* and *Sambucus nigra*, 27 June 2017, M. Kukwa, 19839, 19845, 19849, 19850 (UGDA); Torfowisko Potoczek nature reserve, 54°09'49"N, 16°57'11"E, *Vaccinio uliginosi*-*Betuletum pubescentis*, on wood of log and *Picea abies*, 11 Aug. 2015, M. Kukwa, 16994, 17001a (UGDA); Wysoczyzna Żarnowiecka, Pużyckie Łęgi nature reserve, 54°38'N 17°51'E, *Circaeo*-*Alnetum*, on *Alnus glutinosa* and wood of log, 12 Aug. 2015, M. Kukwa, 17032, 17040, 17053 (UGDA). PORTUGAL. Estremadura, W of Lisbon, WSW of Sintra, WSW of Linhó, along road to Malveira da Serra, nature park Quinta do Pisão, trail in forest with mainly *Pinus* trees, on *Pinus*, 38°45.50'N, 9°24.60'W, 160 m alt., 18 Oct. 2015, P. & B. van den Boom 53789 (hb v.d. Boom); W of Lisbon, S of Sintra, garden of National Palace de la Pena, mixed (mature) trees and shrubs, on tree-fern, 38°47.23'N, 9°23.42'W, 490 m alt., 20 Oct. 2015, P. & B. van den Boom 53907 (hb v.d. Boom). The Netherlands. Noord-Brabant, NE of Oirschot, Woekensesteeg, grid-ref. 51.23.23, trail in mixed forest, on wood of fallen trunk, 4 Oct. 2014, P. & B. van den Boom 51991 (hb v.d. Boom, hb Brand 67113); N of Oirschot, De Mortelen, grid-ref. 51.23.12, trail in damp mixed forest, on *Fagus sylvatica*, 5 June 2017, P. & B. van den Boom 56372 (hb v.d. Boom); E of Best, S side of Wilhelmina channel, grid-ref. 51.24.53, trail in *Pinus* forest, on *Quercus rubra*, 22 July 2018, P. & B. van den Boom 57647 (hb v.d. Boom); ENE of Oostelbeers, Oostelbeerse Heide, grid-ref. 51.32.34, forest, on *Pseudotsuga*, 26 May 2016, P. & B. van den Boom 55028 (hb v.d. Boom); NNW of Wintelre, S side of Straatsche Heide, grid-ref. 51.33.51, *Pinus* forest at edge of *Calluna* heathland with some *Quercus robur* trees, on *Quercus robur*, 14 April 2016, P. & B. van den Boom 54996 (hb v.d. Boom); W of Son, E of Nieuwe Heide, grid-ref. 51.24.45, E side of trail in *Pinus* forest, on *Betula*, 22 June 2014, P. & B. van den Boom 51315 (hb v.d. Boom); S of Best, Aarlesche Heide, S of high-way, grid-ref. 51.33.25, in *Pinus* forest, on *Quercus rubra*, 1 Nov. 2014, P. & B. van den Boom 52515 (hb v.d. Boom); S of Best, Aarlesche Heide, S of highway, grid ref. 51.34.21, grassy *Calluna* heathland, with scattered trees, on *Quercus robur*, 24 Jan. 2014, P. & B. van den Boom 50279 (hb v.d. Boom).

#### Specimen of *Nectriopsis micareae*.

The Netherlands. Noord-Brabant, S of Best, Aarlesche Heide, S of highway, grid ref. 51.34.21, grassy *Calluna* heathland, with scattered trees, on *Micarea microsorediata* growing on *Quercus robur*, 24 Jan. 2014, P. & B. van den Boom 50278 (hb v.d. Boom).

#### Notes.

The new species is morphologically similar to *M. viridileprosa* and *M. soralifera*, but those species differ in their contents of secondary lichen metabolites: *M. viridileprosa* contains gyrophoric acid, whereas *M. soralifera* produces micareic acid (van den Boom and Coppins 2001; Guzow-Krzemińska et al. 2016).

*Micarea microsorediata* produces methoxymicareic acid, a substance present in *M. byssacea*, *M. nigra*, *M. pauli* and other species of the *M. micrococca* clade (Fig. 1), but these species are not sorediate and some of them also have darker apothecia containing the Sedifolia-grey pigment (Czarnota 2007; Czarnota and Guzow-Krzemińska 2010; Launis et al. 2019a; this paper).

### Micarea nigra

Taxon classificationFungiLecanoralesPilocarpaceae

van den Boom, Guzow-Krzemińska, Brand & Sérus. sp. nov.

2950c22c-c2b5-51b5-b09e-6265b5997ca0

MycoBank No.: MB 831825

Fig. 2E

#### Diagnosis.

Species characterised by the production of branched isidia, dark greyish to almost black apothecia containing Cinereorufa-green pigment and measuring 0.15–0.5 mm in diam., (0–)1-septate, narrowly ellipsoidal to clavate ascospores measuring 7.5–12 × (2.5–)3–4.5 µm and the production of methoxymicareic acid.

#### Type.

Portugal. Estremadura, W of Lisbon, W of Sintra, Park de la Monserrate, 200 m alt., 38°47.30'N, 9°25.07'W, parkland with mixed (mature) trees and shrubs, on fern tree, 15 Oct. 2015, P. & B. van den Boom 53726 (holotype LG; isotypes UGDA, hb v.d. Boom, mtSSU GenBank accession number: MK562029).

#### Description.

Thallus inconspicuous, thin, consisting of often branched and vertically proliferating fine isidia; prothallus not seen; areoles up to 0.1 mm in diam.; isidia developing from small areoles, vertically branched and coralloid, in some parts crowded and forming almost a continuous layer, but separated in younger parts of thalli, brownish-green, up to 500 μm tall and 30 μm wide, with a distinct and complete hyphal layer; apothecia abundant, adnate, flat to moderately convex, emarginate, 0.15–0.5 mm in diam., dark greyish to almost black, sometimes with a pale greyish rim; hymenium greenish, with pale brownish streaks, K–, C‒, 30–40 µm tall; epihymenium aeruginose greenish, with Cinereorufa-green pigment, K+ green intensifying; hypothecium hyaline; paraphyses sparse, branched, tips not widened and not pigmented, ca. 1.0‒1.5 µm wide; asci cylindrical to clavate, 24–28 × 9–12 µm, 8-spored; ascospores narrowly ellipsoid to clavate, 7.5–12 × (2.5–)3–4.5 µm, (0–)1-septate; micropycnidia inconspicuous, rare, 30–60 µm in diam., with dark brown top (K–, C‒); microconidia bacilliform, sometimes slightly curved, aseptate, 7–10 × 0.5–0.9 µm; crystalline granules (studied in polarised light) not visible in apothecium, but detected in isidia (sometimes isidia very abundant and sometimes very few), insoluble in K.

Photobiont micareoid, cells thin-walled, 4–8 µm in diam., clustered in compact masses.

#### Chemistry.

Methoxymicareic acid detected by TLC. Cinereorufa-green in apothecia (epihymenium).

#### Habitat and distribution.

Abundantly present on a trunk of a fern tree in a parkland where many tropical and exotic fern and tree species have been introduced.

To date, it is only known from the type locality in Portugal (Sintra).

#### Etymology.

The epithet chosen for this species refers to its very dark appearance, the thallus being dark greenish and the apothecia mostly blackish.

#### Notes.

This species is resolved in the *M. micrococca* group (Fig. 1) and is unique because of its dark grey to almost black apothecia and the presence of Cinereorufa-green pigment in epihymenium.

*Micarea nigra* resembles *M. aeruginoprasina*, *M. isidioprasina* and *M. pauli*. *Micarea aeruginoprasina* and *M. isidioprasina* differ in the presence of micareic acid instead of methoxymicareic acid and paler apothecia. In addition, *M. aeruginoprasina* produces different pigment in the apothecia (Sedifolia-grey). *Micarea pauli* differs in the production of methoxymicareic acid, Sedifolia-grey pigment in the apothecia and different distribution (see under that species).

Some morphs of *M. prasina* can also have dark apothecia, but this species contains micareic acid and Sedifolia-grey in the apothecia (Coppins 1983; Czarnota 2007; Launis et al. 2019a, b). *Micarea subviridescens* can have blackish apothecia and is sometimes epiphytic, but it produces prasinic acid (Coppins 1983).

### Micarea pauli

Taxon classificationFungiLecanoralesPilocarpaceae

Guzow-Krzemińska, Łubek & Kukwa sp. nov.

ae39cde9-b11a-5832-af79-851cf1d56387

MycoBank No.: MB 831826

Fig. 2F

#### Diagnosis.

Species characterised by isidiate thallus, pale grey to grey beige apothecia with Sedifolia-grey pigment, 0–1-septate, ovoid, ellipsoidal or oblong ascospores measuring 7–13 × 3.5–4.5 μm and the presence of methoxymicareic acid.

#### Type.

Poland. Równina Bielska, Białowieża Forest, Białowieża National Park, forest section no 256, *Carici elongatae*-*Alnetum*, on *Alnus glutinosa*, 17 Aug 2015, M. Kukwa & 17240, A. Łubek (holotype UGDA; isotype KTC, mtSSU GenBank accession number: MK562014, *Mcm7* GenBank accession number: MN105912).

#### Description.

Thallus crustose, indeterminate, endosubstratal to rarely episubstratal in non-isidiate parts and then as a thin greenish film over the substrate or minutely areolate, isidiate; prothallus not evident; areoles up to 0.1 mm in diam., green, soon developing isidia; isidia branched and coralloid, crowded and forming almost a continuous layer over the substrate, but separated in younger parts of thalli, green to olive green, up to 0.5 mm tall and 30 μm wide, with a distinct and complete hyphal layer; apothecia rarely developed (in 2 specimens only), beige with spots of grey pigment, pale grey to grey-beige, up to 0.5 mm in diam., irregular in shape, convex, with a white rim; excipulum as a narrow, hyaline zone, hyphae radiating, branched and anastomosing; hymenium up to 45 μm tall; epihymenium partly olive-grey due to the presence of Sedifolia-grey pigment (K+ violet, C+ violet) confined to the gel matrix; hypothecium hyaline to pale straw coloured in upper part; paraphyses 1–1.5 μm thick, sparse, mostly apically branched and anastomosing, hyaline throughout; asci cylindrical-clavate, 30–35 × 9–12 μm, 8-spored; ascospores, 0–1-septate, ovoid, ellipsoidal or oblong, 7–13 × 3.5–4.5 μm; pycnidia not seen; crystalline granules (studied in polarised light) abundant in hymenium and isidia, soluble in K.

Photobiont chlorococcoid, micareoid, cells globose to ellipsoidal, 4–7 μm in diam.

#### Chemistry.

Methoxymicareic acid detected by TLC. Sedifolia-grey in apothecia (epihymenium).

#### Habitat and distribution.

This species is so far known only in Poland from Białowieża Forest, where it grows in deciduous forests on bark of *Alnus glutinosa* (5 specimens), *Tilia cordata* (1 specimen) and on wood (2 specimens).

#### Etymology.

The species is named after our friend, Paweł Czarnota, specialist in the genus who monographed it in Poland.

#### Additional specimens examined.

Poland. Równina Bielska, Białowieża Forest; Białowieża National Park, forest section no 256, *Carici elongatae*-*Alnetum*, on *Alnus glutinosa* and *Picea abies*, Aug. 2014, M. Kukwa 13194, 13330, 13345, A. Łubek (KTC, UGDA); ibidem, *Tilio-Carpinetum*, on *Tilia cordata*, Aug. 2014, M. Kukwa 14101, A. Łubek (KTC, UGDA); ibidem, *Carici elongatae*-*Alnetum*, on *Alnus glutinosa*, 17 Aug. 2015, M. Kukwa, 17227, A. Łubek (KTC, UGDA, hb v.d. Boom); ibidem, *Querco*-*Piceetum*, on *Alnus glutinosa*, 29 Sept. 2015, M. Kukwa 17544, A. Łubek (KTC, UGDA); ibidem, *Peucedano*-*Pinetum*, on *Alnus glutinosa*, March 2015, M. Kukwa 13308 (KTC, UGDA); *Pino-Quercetum*, on wood of snag, 1 Oct. 2015, M. Kukwa 17582a, A. Łubek (KTC); *Pino-Quercetum*, on wood of log, 2 Oct. 2015, M. Kukwa 17619, A. Łubek (KTC, UGDA); *Carici elongatae*-*Alnetum*, on *Alnus glutinosa*, 3 Oct. 2015, M. Kukwa 17621, A. Łubek (KTC, UGDA).

#### Notes.

*Micarea pauli* is an isidiate species with Sedifolia-grey pigment in its apothecia. It can be separated from the similar *M. isidioprasina*, with which it grows in Białowieża Forest, by the presence of methoxymicareic acid.

*Micarea aeruginoprasina* and *M. nigra* are also similar in thallus morphology, but they differ in the pigmentation of apothecia. *Micarea aeruginoprasina* develops pale cream to pale brown or aeruginose apothecia, which are often mottled in colour in one apothecium, whereas in *M. nigra* the apothecia are dark greyish to black. Without apothecia, they can be difficult to separate from *M. pauli*, especially *M. nigra* which also contains methoxymicareic acid (*M. aeruginoprasina* produces micareic acid), but so far, *M. aeruginoprasina* and *M. nigra* are only known from the Azores and continental Portugal, respectively.

Methoxymicareic acid is the main secondary metabolite, also found in *M. byssacea*, *M. micrococca* and other species in the *M. micrococca* clade (Fig. 1), but those species are never isidiate (Czarnota 2007; Czarnota and Guzow-Krzemińska 2010; Launis et al. 2019a).

## Supplementary Material

XML Treatment for Micarea aeruginoprasina

XML Treatment for Micarea azorica

XML Treatment for Micarea isidioprasina

XML Treatment for Micarea microsorediata

XML Treatment for Micarea nigra

XML Treatment for Micarea pauli

## Supplementary materials

Supplementary material 1 Figures S1–S15 presenting ancestral character state reconstructions Data type: PDF file https://binary.pensoft.net/file/321988This dataset is made available under the Open Database License (http://opendatacommons.org/licenses/odbl/1.0/). The Open Database License (ODbL) is a license agreement intended to allow users to freely share, modify, and use this Dataset while maintaining this same freedom for others, provided that the original source and author(s) are credited.Beata Guzow-Krzemińska, Emmanuël Sérusiaux, Pieter P. G. van den Boom, A. Maarten Brand, Annina Launis, Anna Łubek, Martin Kukwa

Supplementary material 2 Tables S1–S4 Data type: PDF file Explanation note: Additional tables with list of specimens used in this study, data matrices for ancestral character state reconstructions and probabilities of reconstructions with model Mk1. https://binary.pensoft.net/file/321989This dataset is made available under the Open Database License (http://opendatacommons.org/licenses/odbl/1.0/). The Open Database License (ODbL) is a license agreement intended to allow users to freely share, modify, and use this Dataset while maintaining this same freedom for others, provided that the original source and author(s) are credited.Beata Guzow-Krzemińska, Emmanuël Sérusiaux, Pieter P. G. van den Boom, A. Maarten Brand, Annina Launis, Anna Łubek, Martin Kukwa
