# Supplementary material for: Understanding the evolution of phenotypical characters in the Micarea prasina group (Pilocarpaceae) and descriptions of six new species within the group
Source: MycoKeys. 2019 Jul 31;57:1–30. doi: 10.3897/mycokeys.57.33267 (PMC6685933; doi:10.3897/mycokeys.57.33267)
Supplement: Supplementary material 1 [file mycokeys-57-001-s002.pdf]

Supplementary material 1 Ancestral character state reconstructions

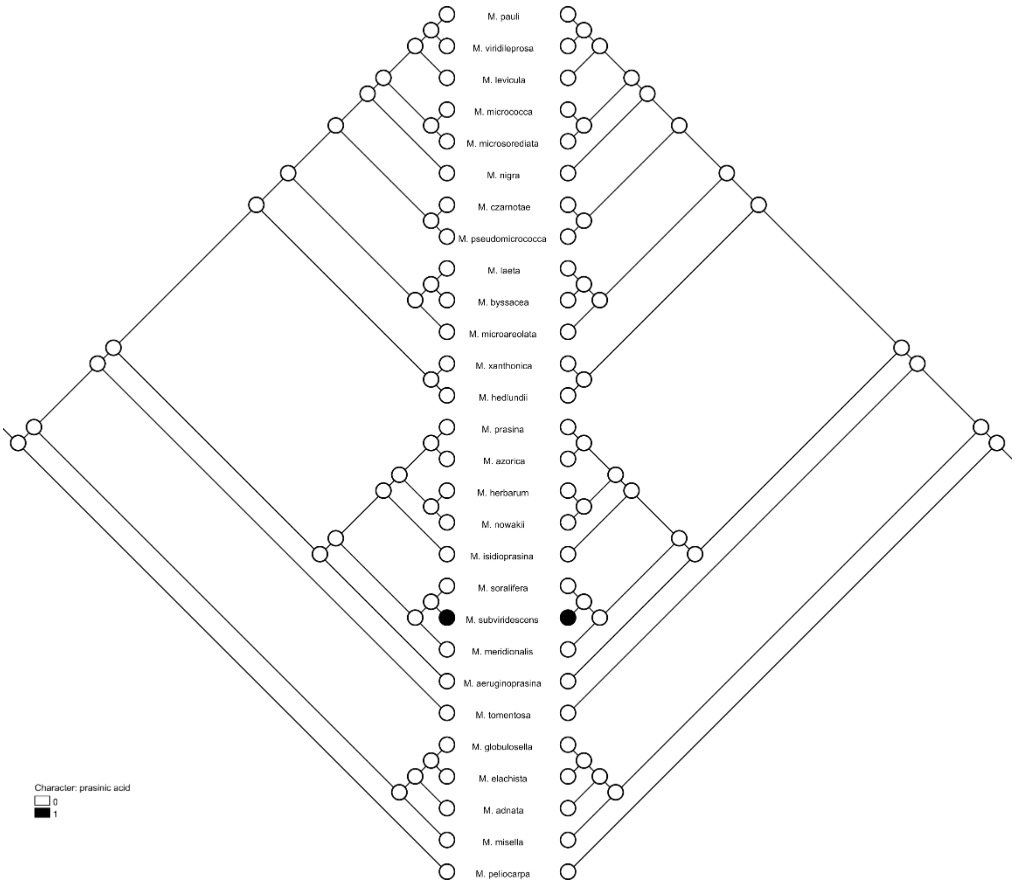

Figure S1. Maximum parsimony (left panel) and maximum likelihood (right panel) reconstructions of prasinic acid production based on the Bayesian tree performed in Mesquite v. 3.5. The estimated proportional likelihoods of alternative ancestral states are indicated by pie charts on the corresponding nodes (0 – absent, 1 – present).

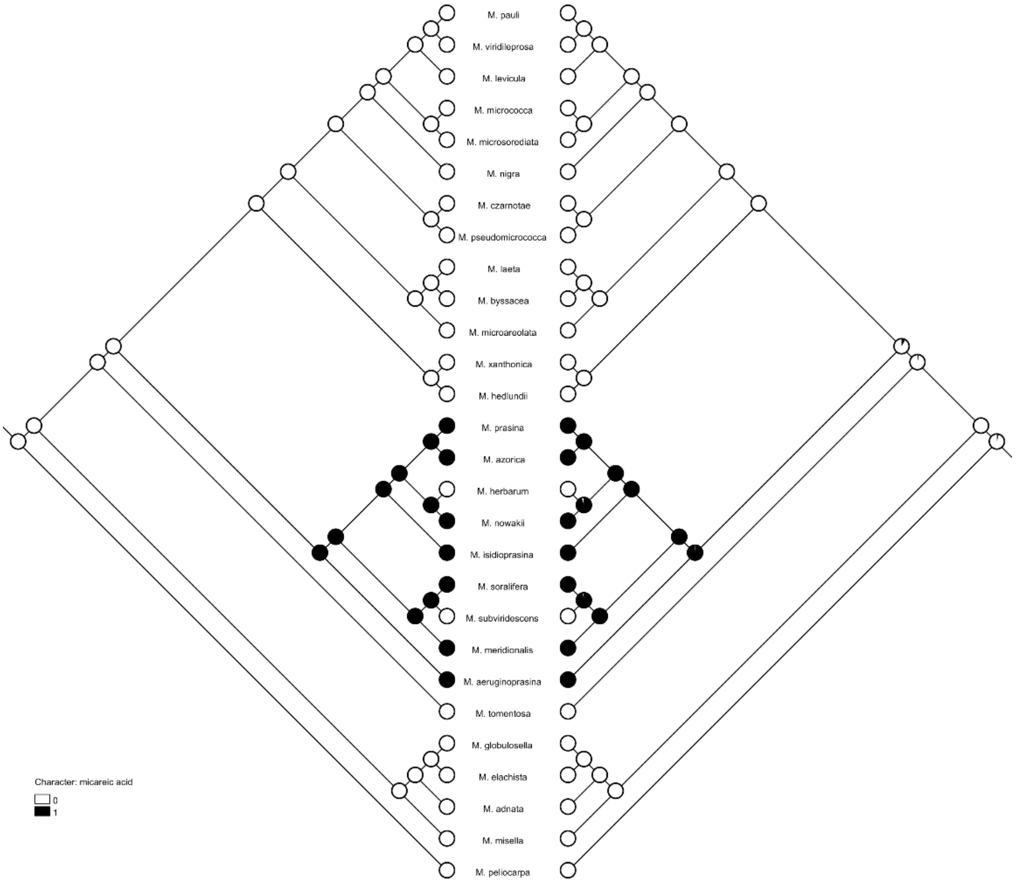

Figure S2. Maximum parsimony (left panel) and maximum likelihood (right panel) ancestral state reconstructions of micareic acid production based on the Bayesian tree performed in Mesquite v. 3.5. The estimated proportional likelihoods of alternative ancestral states are indicated by pie charts on the corresponding nodes (0 – absent, 1 – present).

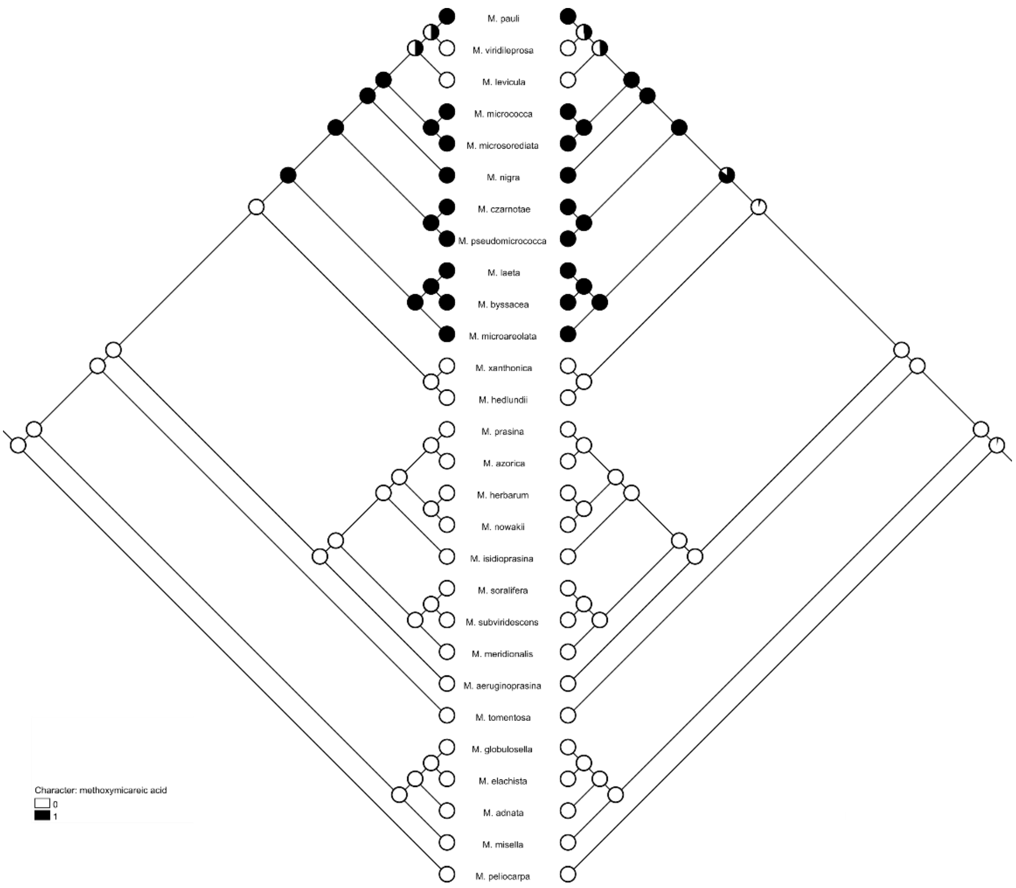

Figure S3. Maximum parsimony (left panel) and maximum likelihood (right panel) ancestral state reconstructions of methoxymicareic acid production based on the Bayesian tree performed in Mesquite v. 3.5. The estimated proportional likelihoods of alternative ancestral states are indicated by pie charts on the corresponding nodes (0 – absent, 1 – present).

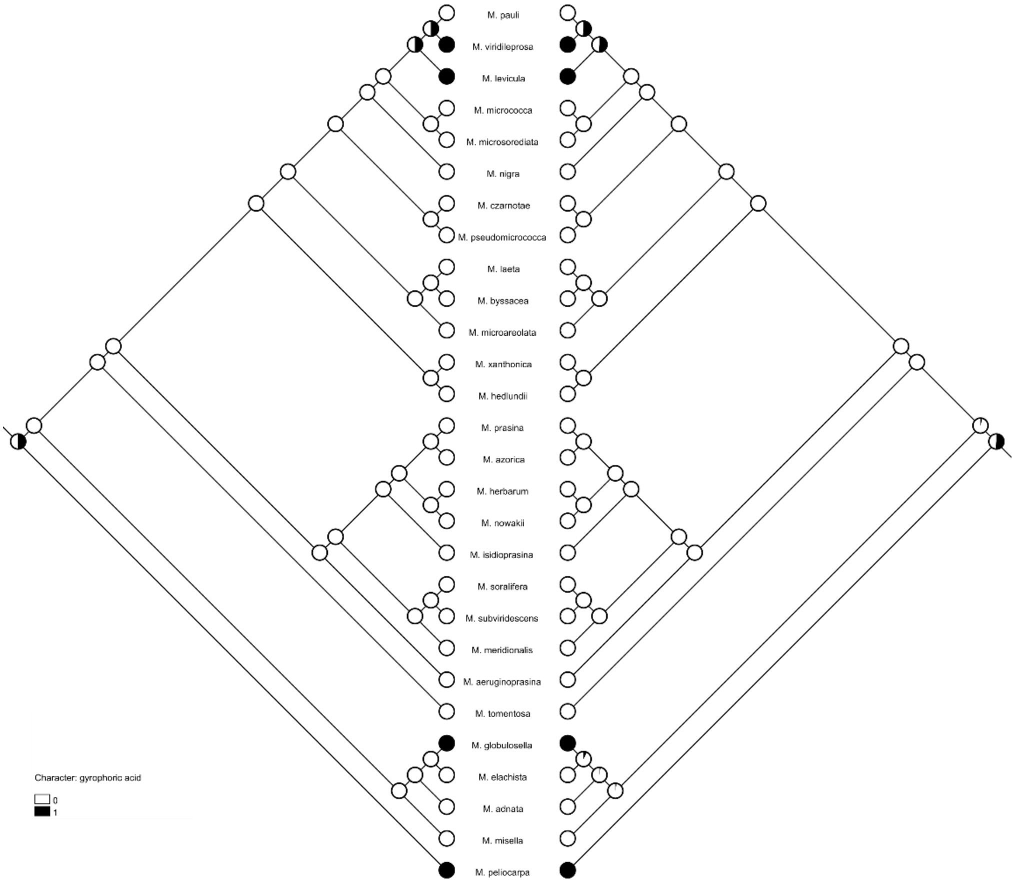

Figure S4. Maximum parsimony (left panel) and maximum likelihood (right panel) ancestral state reconstructions of gyrophoric acid production based on the Bayesian tree performed in Mesquite v. 3.5. The estimated proportional likelihoods of alternative ancestral states are indicated by pie charts on the corresponding nodes (0 – absent, 1 – present).

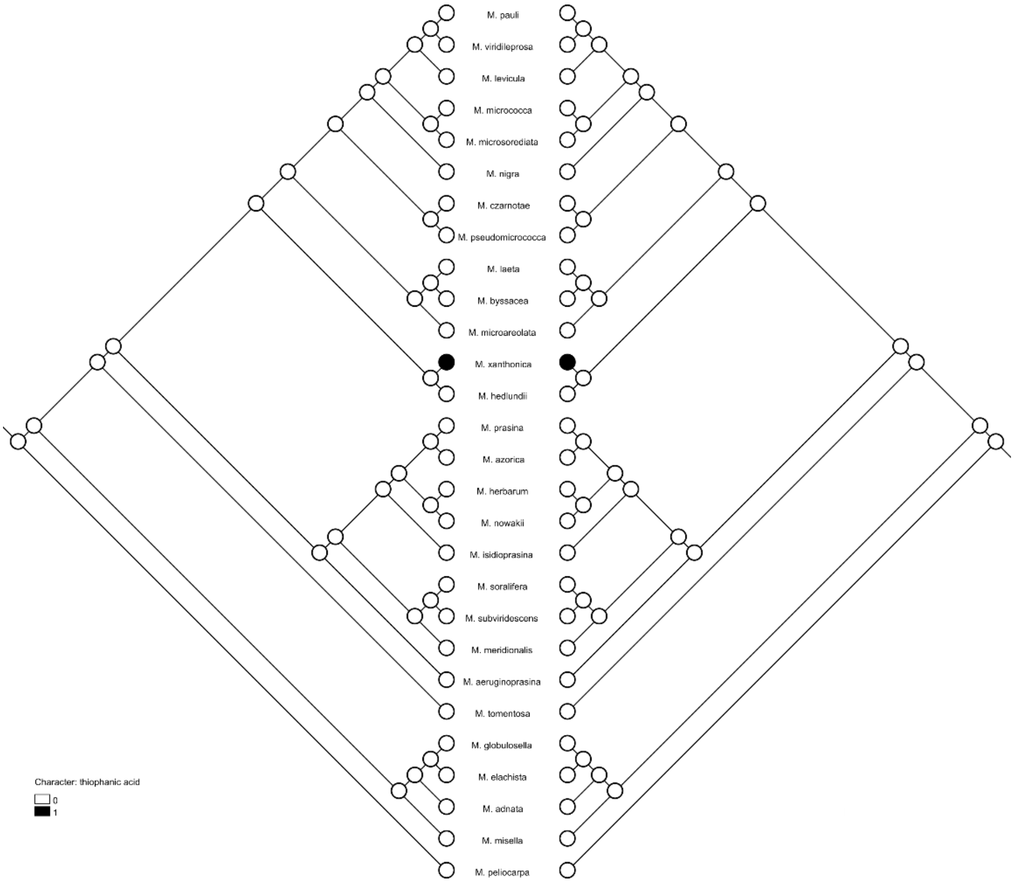

Figure S5 Maximum parsimony (left panel) and maximum likelihood (right panel) ancestral state reconstructions of thiophanic acid production based on the Bayesian tree performed in Mesquite v. 3.5. The estimated proportional likelihoods of alternative ancestral states are indicated by pie charts on the corresponding nodes (0 – absent, 1 – present).

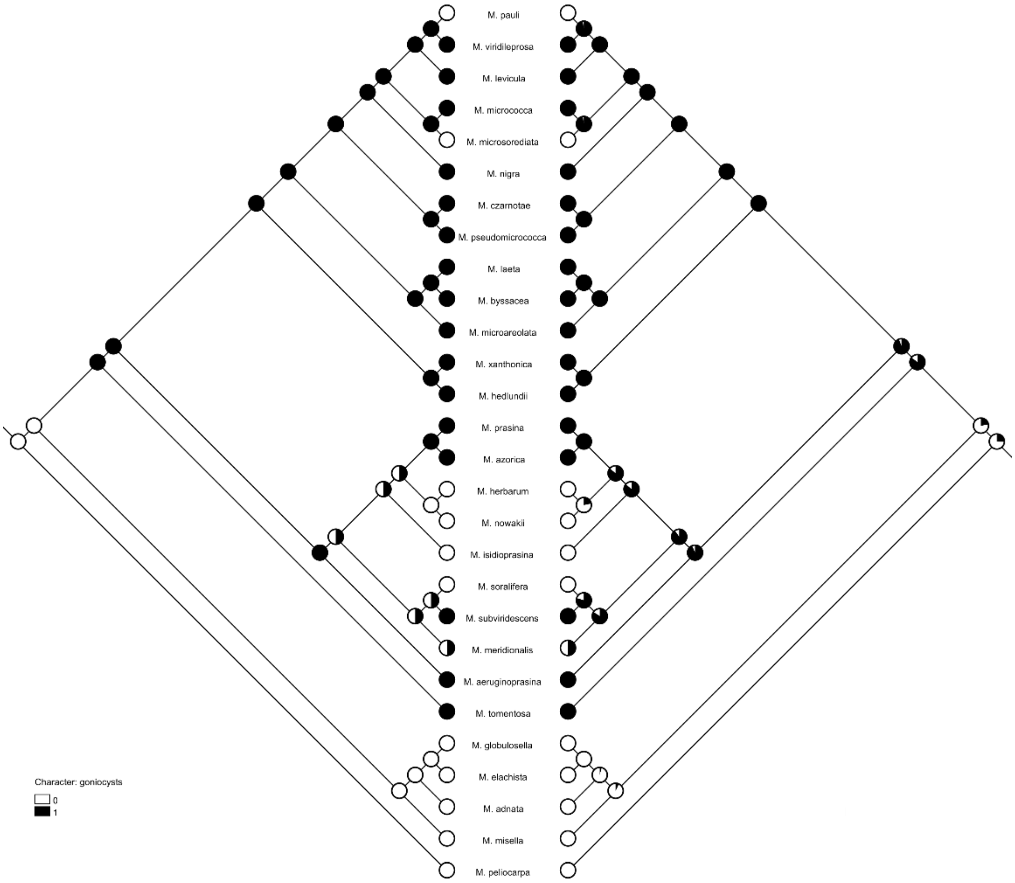

Figure S6. Maximum parsimony (left panel) and maximum likelihood (right panel) ancestral state reconstructions of the presence pf goniocysts based on the Bayesian tree performed in Mesquite v. 3.5. The estimated proportional likelihoods of alternative ancestral states are indicated by pie charts on the corresponding nodes (0 – absent, 1 – present).

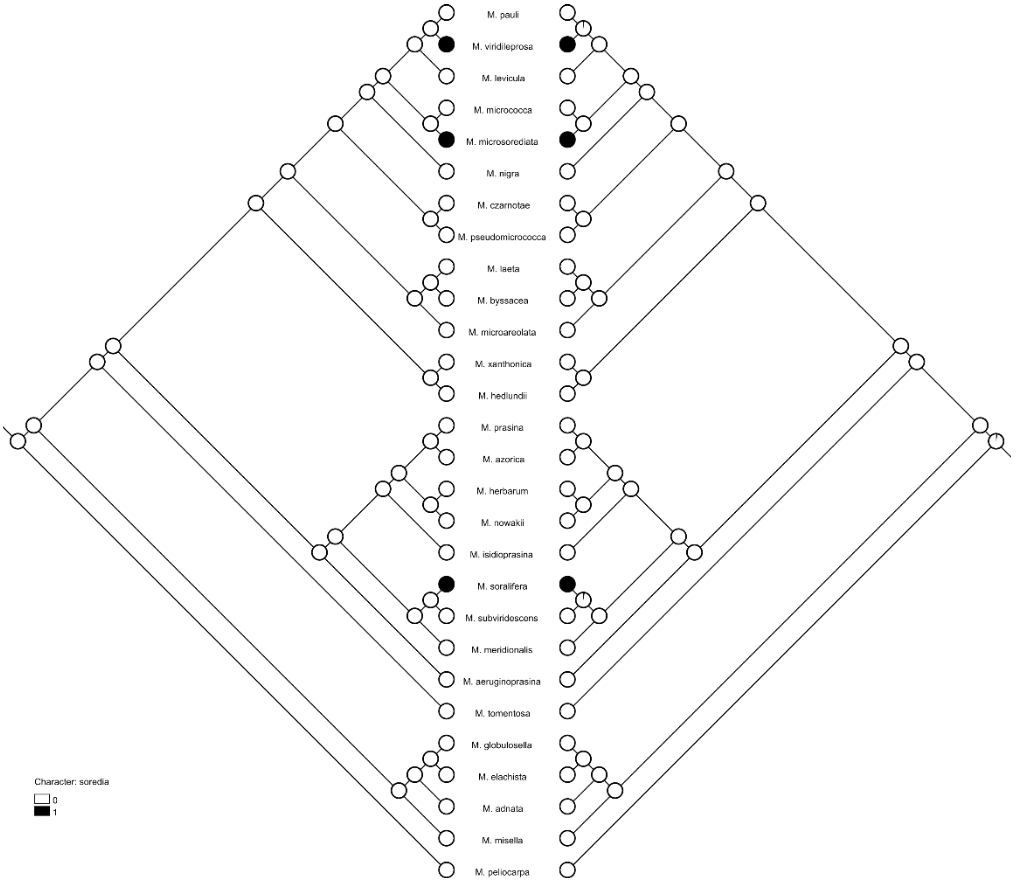

Figure S7. Maximum parsimony (left panel) and maximum likelihood (right panel) ancestral state reconstructions of the presence of soredia based on the Bayesian tree performed in Mesquite v. 3.5. The estimated proportional likelihoods of alternative ancestral states are indicated by pie charts on the corresponding nodes (0 – absent, 1 – present).

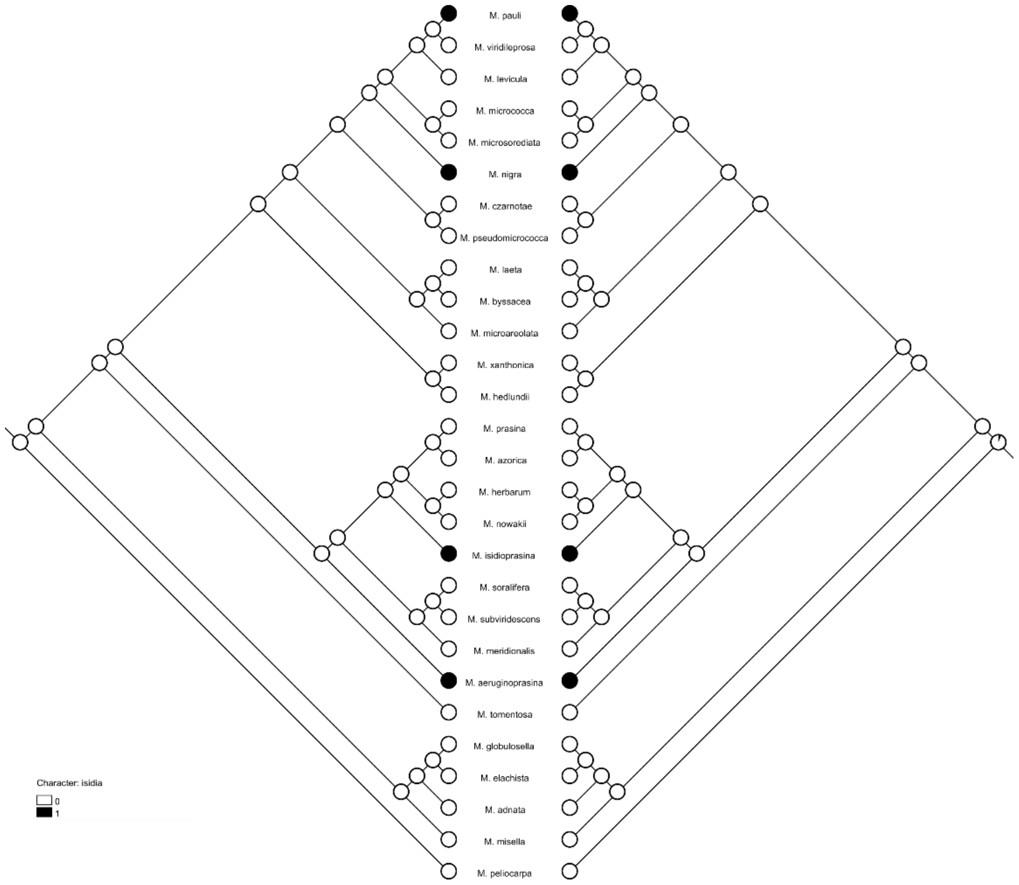

Figure S8. Maximum parsimony (left panel) and maximum likelihood (right panel) ancestral state reconstructions of the presence of isidia based on the Bayesian tree performed in Mesquite v. 3.5. The estimated proportional likelihoods of alternative ancestral states are indicated by pie charts on the corresponding nodes (0 – absent, 1 – present).

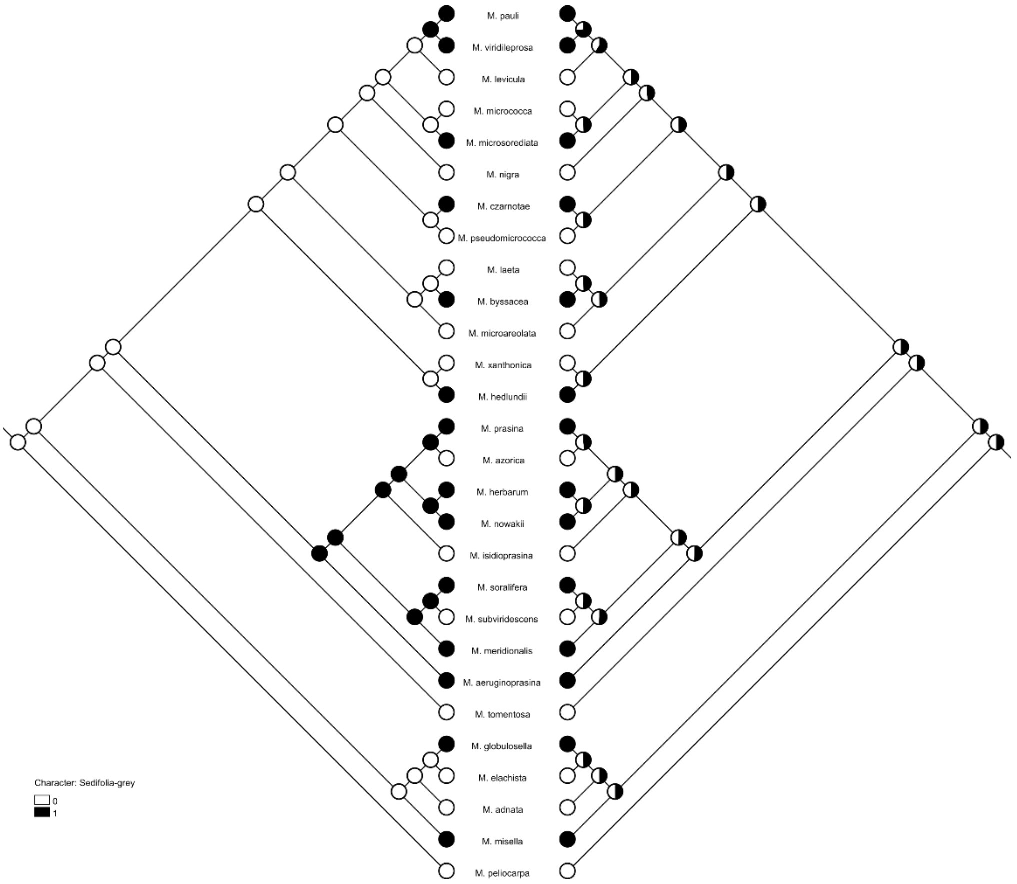

Figure S9. Maximum parsimony (left panel) and maximum likelihood (right panel) ancestral state reconstructions of Sedifolia-grey production based on the Bayesian tree performed in Mesquite v. 3.5. The estimated proportional likelihoods of alternative ancestral states are indicated by pie charts on the corresponding nodes (0 – absent, 1 – present).

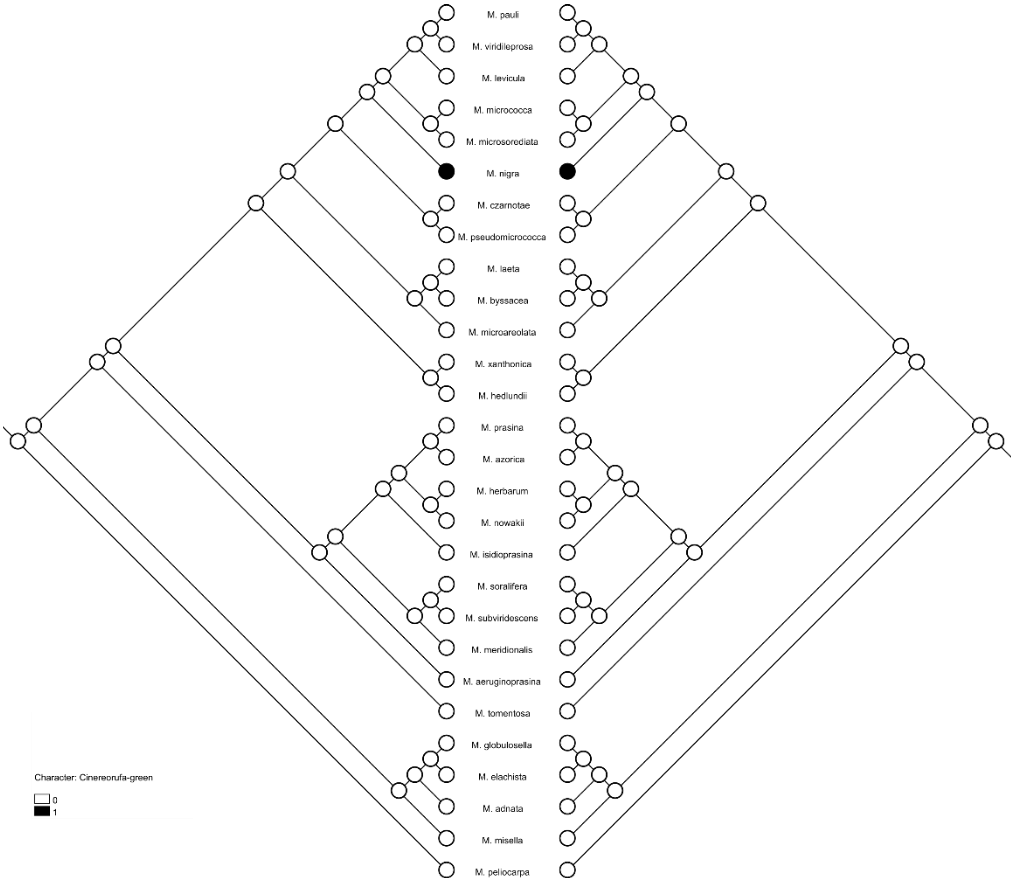

Figure S10. Maximum parsimony (left panel) and maximum likelihood (right panel) ancestral state reconstructions of Cinereorufa-green production based on the Bayesian tree performed in Mesquite v. 3.5. The estimated proportional likelihoods of alternative ancestral states are indicated by pie charts on the corresponding nodes (0 – absent, 1 – present).

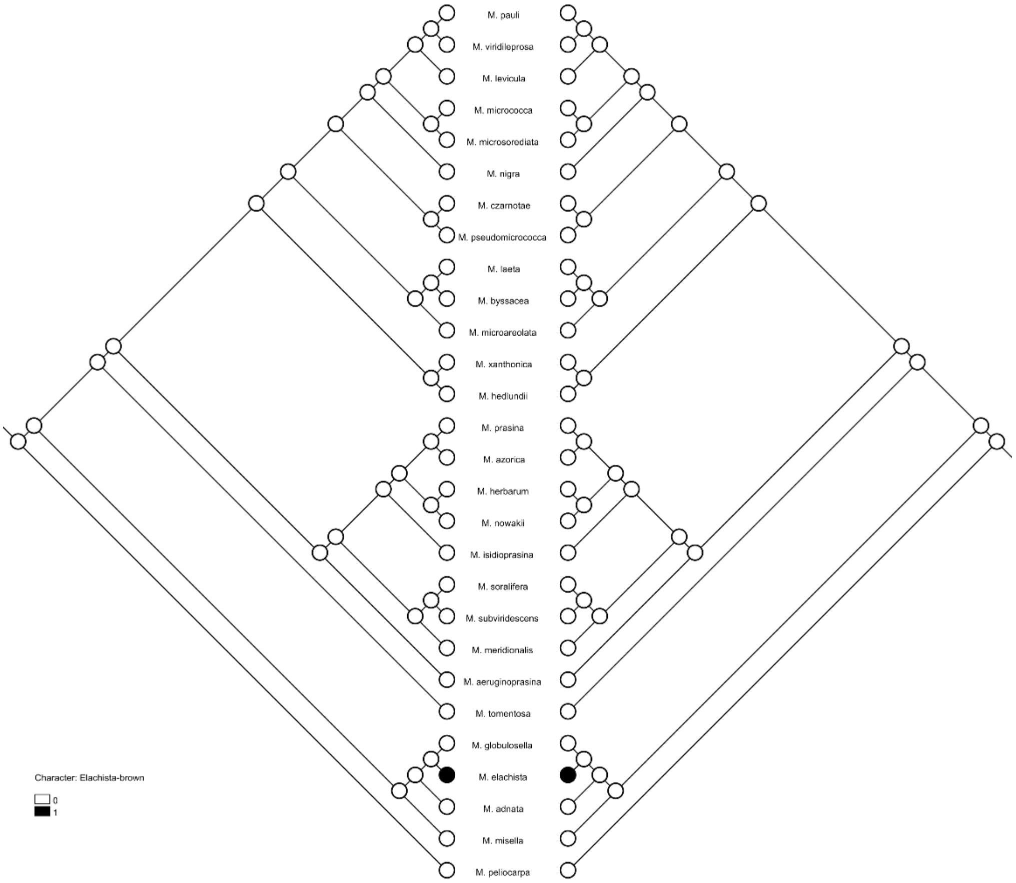

Figure S11. Maximum parsimony (left panel) and maximum likelihood (right panel) ancestral state reconstructions of Elachista-Brown production based on the Bayesian tree performed in Mesquite v. 3.5. The estimated proportional likelihoods of alternative ancestral states are indicated by pie charts on the corresponding nodes (0 – absent, 1 – present).

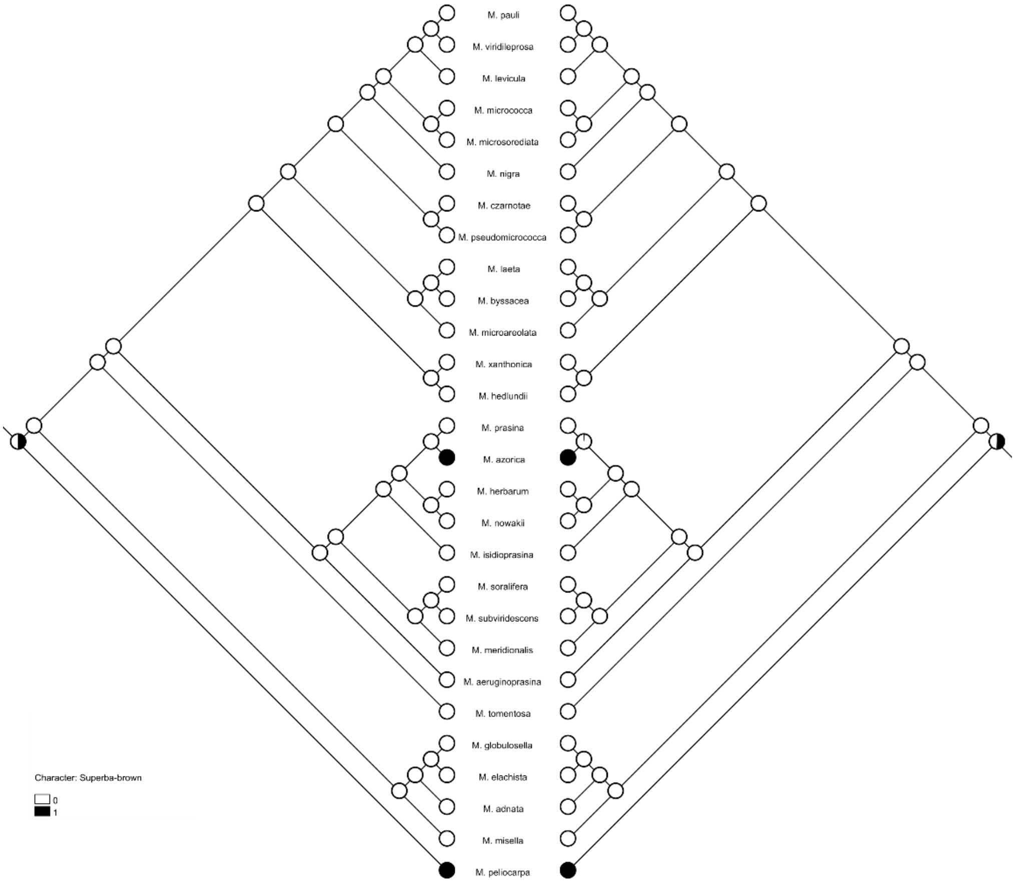

Figure S12. Maximum parsimony (left panel) and maximum likelihood (right panel) ancestral state reconstructions of Superba-brown production based on the Bayesian tree performed in Mesquite v. 3.5. The estimated proportional likelihoods of alternative ancestral states are indicated by pie charts on the corresponding nodes (0 – absent, 1 – present).

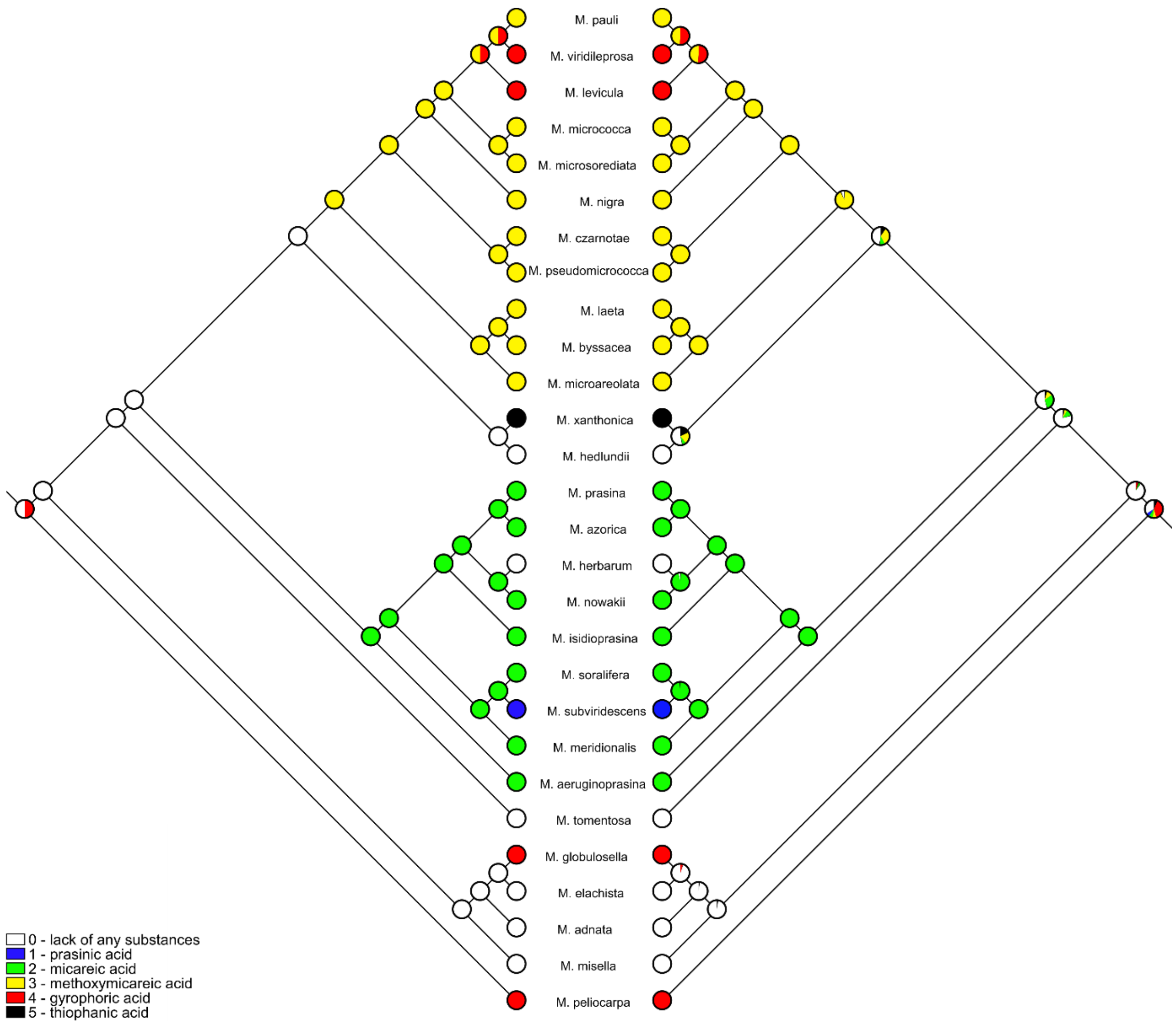

Figure S13. Ancestral character state reconstructions of secondary metabolites production. Maximum parsimony (left panel) and maximum likelihood (right panel) reconstructions of secondary metabolites production (i.e. prasinic, micareic, methoxymicareic, gyrophoric and thiophanic acids) in the *Micarea prasina* group based on the Bayesian tree and performed in Mesquite v. 3.5. The estimated proportional likelihoods of alternative ancestral states are indicated by pie charts on the corresponding nodes.

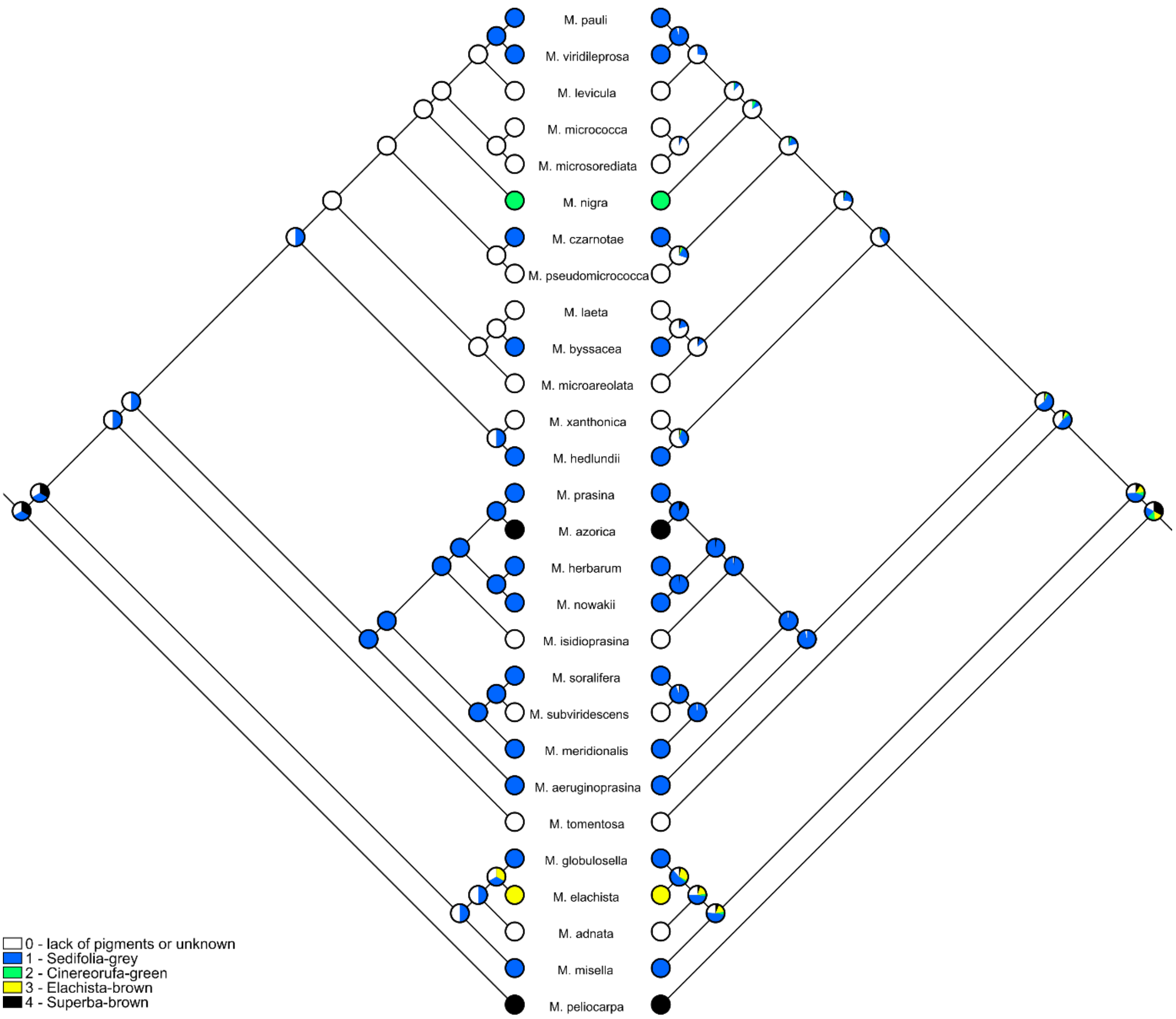

Figure S14. Ancestral character state reconstructions of apothecial pigmentation. Maximum parsimony (left panel) and maximum likelihood (right panel) reconstructions of apothecial pigmentation (i.e. Sedifolia-grey, Cinereorufa-green, Elachista-brown, Superba-brown) in the *Micarea prasina* group based on the Bayesian tree and performed in Mesquite v. 3.5. The estimated proportional likelihoods of alternative ancestral states are indicated by pie charts on the corresponding nodes.

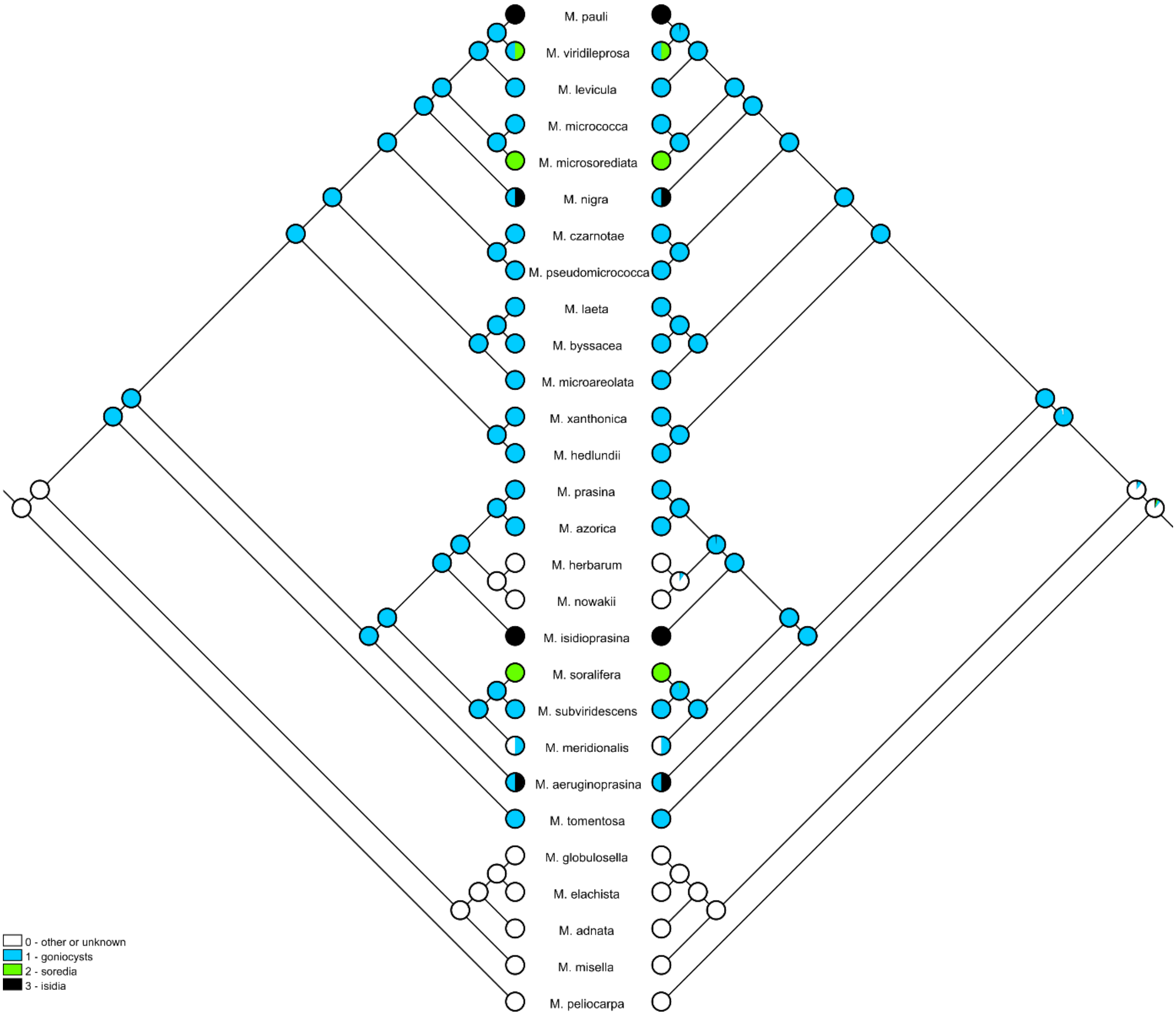

Figure S15. Ancestral character state reconstructions of selected morphological characters. Maximum parsimony (left panel) and maximum likelihood (right panel) reconstructions of selected morphological characters, i.e. goniocysts, soredia and isidia, in the *Micarea prasina* group based on the Bayesian tree and performed in Mesquite v. 3.5. The estimated proportional likelihoods of alternative ancestral states are indicated by pie charts on the corresponding nodes.
